# Scalable integration of multiomic single-cell data using generative adversarial networks

**DOI:** 10.1093/bioinformatics/btae300

**Published:** 2024-05-02

**Authors:** Valentina Giansanti, Francesca Giannese, Oronza A Botrugno, Giorgia Gandolfi, Chiara Balestrieri, Marco Antoniotti, Giovanni Tonon, Davide Cittaro

**Affiliations:** Department of Informatics, Systems and Communication, Università degli Studi di Milano-Bicocca, Milan, 20125, Italy; Center for Omics Sciences, IRCCS San Raffaele Scientific Institute, Milan, 20132, Italy; Center for Omics Sciences, IRCCS San Raffaele Scientific Institute, Milan, 20132, Italy; Functional Genomics of Cancer Unit, IRCCS San Raffaele Scientific Institute, Milan, 20132, Italy; Università Vita-Salute San Raffaele, Milan, 20132, Italy; Center for Omics Sciences, IRCCS San Raffaele Scientific Institute, Milan, 20132, Italy; Center for Omics Sciences, IRCCS San Raffaele Scientific Institute, Milan, 20132, Italy; Experimental Hematology Unit, IRCCS San Raffaele Scientific Institute, Milan, 20132, Italy; Department of Informatics, Systems and Communication, Università degli Studi di Milano-Bicocca, Milan, 20125, Italy; Bicocca Bioinformatics Biostatistics and Bioimaging Centre-B4, Università degli Studi di Milano-Bicocca, Milan, 20125, Italy; Istituto di Bioimmagini e Fisiologia Molecolare, Consiglio Nazionale delle Ricerche (CNR), Milan, 20090, Italy; Center for Omics Sciences, IRCCS San Raffaele Scientific Institute, Milan, 20132, Italy; Functional Genomics of Cancer Unit, IRCCS San Raffaele Scientific Institute, Milan, 20132, Italy; Università Vita-Salute San Raffaele, Milan, 20132, Italy; Center for Omics Sciences, IRCCS San Raffaele Scientific Institute, Milan, 20132, Italy

## Abstract

**Motivation:**

Single-cell profiling has become a common practice to investigate the complexity of tissues, organs, and organisms. Recent technological advances are expanding our capabilities to profile various molecular layers beyond the transcriptome such as, but not limited to, the genome, the epigenome, and the proteome. Depending on the experimental procedure, these data can be obtained from separate assays or the very same cells. Yet, integration of more than two assays is currently not supported by the majority of the computational frameworks avaiable.

**Results:**

We here propose a Multi-Omic data integration framework based on Wasserstein Generative Adversarial Networks suitable for the analysis of paired or unpaired data with a high number of modalities (>2). At the core of our strategy is a single network trained on all modalities together, limiting the computational burden when many molecular layers are evaluated.

**Availability and implementation:**

Source code of our framework is available at https://github.com/vgiansanti/MOWGAN

## 1 Introduction

The availability of single-cell sequencing technologies made easier the understanding of the complex organization of tissues and organs and the interplay among different types of cells. Here, cell properties can be characterized at different layers, in terms of transcriptome (Klein *et al.* 2015, Macosko *et al.* 2015, Rosenberg *et al.* 2018, Clark *et al.* 2023), genome (Wang *et al.* 2012, Zong *et al.* 2012, Chen *et al.* 2017), epigenome (Rotem *et al.* 2015, Clark *et al.* 2018, Bartosovic *et al.* 2021), and proteome (Stoeckius *et al.* 2017, Gebreyesus *et al.* 2022). Depending on the experimental procedure, these data are available from the same cells or from separate assays (Zhu *et al.* 2020). Multi-omic data from the same cells are currently limited to up to two-third modalities (or layers), but the prospective is to have a higher rate of co-observation (Ogbeide *et al.* 2022, Vandereyken *et al.* 2023), as long as additional assays do not compromise profiling of other layers, keeping the cell intact.

Proper integration of multiomic data is one of the grand challenges in single-cell data analysis (Lähnemann *et al.* 2020). Integration is considered a difficult task as it requires specific computational models supporting multidimensional data, it is based on generally unknown models of mutual dependence and causal relationships among modalities, and it includes all the analysis challenges that are peculiar to each single modality (e.g. sampling bias, high sparsity).

Data integration links different data sources to derive a more comprehensive and biologically meaningful description of the object under analysis. This task can be addressed in three different ways depending on the type of available anchors used to combine multiple data sources (Argelaguet *et al.* 2021): we refer to *horizontal integration* when integration is performed between different datasets representing the same data type (i.e., multiple scRNA-seq dataset) collected from multiple samples at different locations or time points. *Vertical integration* is performed when more modalities are assayed from the same cell, for example epigenome and transcriptome (Clark *et al.* 2018, Chen, Lake and Zhang, 2019, Ma *et al.* 2020), transcriptome and proteins (Stoeckius *et al.* 2018), genome and transcriptome (Macaulay *et al.* 2015), or any other combination (Hou *et al.* 2016, Swanson *et al.* 2021, Tedesco *et al.* 2022, Meers *et al.* 2023). Vertical integration is challenging both from an experimental and an interpretative point of view. Finally, we refer to *diagonal integration* if both cells and features are different for all datasets. This last scenario may be considered the hardest to solve and yet it is possibly the most common, given the pace at which single-cell datasets are produced (Svensson *et al.* 2018).

While horizontal integration is essentially a problem of batch correction (Tran *et al.* 2020), integration of multimodal data requires solving the vertical and/or diagonal settings. To the best of our knowledge, a fully integrated and generalizable way to analyze such data is still missing. The main solutions proposed so far refer to *Manifold Alignment* (MA) applications and *Deep Learning* (DL) (Liu *et al.* 2019, Dou *et al.* 2020, Stark *et al.* 2020, Ahmed *et al.* 2021, Cao, Hong and Wan, 2021, Gong *et al.* 2021, Minoura *et al.* 2021, Wangwu *et al.* 2021, Zuo and Chen, 2021, Amodio *et al.* 2022, Cao *et al.* 2022, Demetci *et al.* 2022a, 2022b, Xu et al. 2022, Zhao *et al.* 2022). Both approaches share the final goal of representing multiple feature sets in a common manifold embedding. MA methods try to find a common latent space (manifold) to describe the data, while DL methods develop networks specific for the single omic and work on their embeddings (i.e. learned low-dimensional representations of the given data).

In order to produce their results, both MA and DL methods must assume some constraints. Restrictive assumptions are generally applied on the data, like correspondences between the features (e.g. converting chromatin accessibility data into gene activity scores; Granja *et al.* 2021) and/or between cells, or assumptions on the data distribution. These requirements are difficult to fulfill and to generalize, making them unfit for datasets where no prior knowledge is available. Furthermore, they are typically considered to address the integration of only two molecular layers (e.g. RNA and ATAC), so scaling to three or more modalities could be impractical.

In addition to technical limitations and experimental costs, many large datasets have been made available for single omics only (Regev *et al.* 2017, Bock *et al.* 2021, Zhang *et al.* 2021). This urges the need for the development of an approach to integrate paired or unpaired data which is also free from assumptions and flexible with respect to the number of omics available. To address these issues, we propose MOWGAN, a DL framework for the analysis of Multi-Omics paired or unpaired data based on Wasserstein Generative Adversarial Networks (MOWGAN). Our approach is designed to accommodate any kind and number of single-cell assays without priors on the relationships among the inputs. MOWGAN learns the structure of single assays and infers the optimal couplings between pairs of assays. In doing so, MOWGAN generates synthetic multiomic datasets that can be used to transfer information among the measured assays by bridging (Hao *et al.* 2024). We benchmarked MOWGAN with existing methods for multimodal single-assay data integration on various single-cell datasets and showed how it could be used to unveil hidden biology. We show that MOWGAN generates much more reliable results when looking at the cell type annotation shared between the layers.

## 2 Methods

### 2.1 WGAN-GP architecture

MOWGAN core component is a WGAN-GP composed of two networks, a *generator* and a *critic*. The *generator* is designed with three *convolutional 1D* layers (Conv1D) and two *batch normalization* layers (BN). The *critic* is designed with two Conv1D layers and a single Dense layer with one unit. All Conv1D layers have common parameters: *kernel size *=* *2, *stride *=* *1, *activation function* = ReLu. The number of *filters* for Conv1D layer changes with the network depth: default number of filters for the *generator* are 512, 128, and 20, for the *critic* 128 and 512.

Subnetwork uses different optimizers: Adam optimizer for the *generator* with *learning rate *=* *0.001, *beta_1 *=* *0.5, *beta_2 *=* *0.9, *epsilon *=* *1e-07; RMSprop optimizer with *learning rate *=* *0.0005 in the *critic*.

### 2.2 Mini-batch formation

To calculate the first component of the Laplacian Eigenmap we used scikit’s *sklearn.manifold.SpectralEmbedding* class (*n_components *=* *1, *affinity* = ‘precomputed’). The input to SpectralEmbedding was the weighted kNN graph that is calculated by scanpy’s *sc.pp.neighbors* function during data processing. The resulting vector is used to sort the data embedding used to train MOWGAN (e.g. Principal Component Analysis (PCA)).

During WGAN-GP training, *N* random cells from the first modality are chosen to form a source mini-batch (*N *=* *256). The embedding (*X*) and the Eigenmap vector (*y*) are used to train a Bayesian ridge regressor *R* using scikit’s *sklearn.linear_model.BayesianRidge* class. Next, 50 candidate mini-batches made by *N *=* *256 cells are randomly selected from the second modality. Each target is scored using the *R* regressor on the target *X* and *y* values. The highest scoring mini-batch is coupled with the source mini-batch and presented to the WGAN-GP.

In the case of paired, multimodal data, this pre-match step can be skipped; cells sharing the same barcode in all modalities are sorted lexicographically and mini-batches are formed sampling cells having the same index in the embedding matrix.

### 2.3 Grid search

We applied a grid search policy to test the dependency of MOWGAN to hyperparameters. We tested multiple filters *f* = {8, 32, 64, 128, 256, 512} in the Conv1D layers, and number of components selected from the embeddings *C* = {5,10,15}. We also set *C* as the number of filters in the *generator’*s hidden layer. A total of 216 models were trained, with training time for a single model of approximately 3 h every 100 000 epochs. Fifty-one models returned NaNs in the loss function, one model produced data collapsed in a single point.

### 2.4 Feature reconstruction

MOWGAN outputs synthetic cells projected in the same embedding of cells used for training, but not their features (i.e. genes or regions). Therefore, we fit source features to embeddings using a kNN-regressor (*sklearn.neighbors.KNeighborsRegressor*, *n_neighbors *=* *2) on each modality. The regressors are then used to predict the feature values of synthetic cells.

### 2.5 Peripheral blood mononuclear cell datasets

Public data for peripheral blood mononuclear cell (PBMC) were downloaded from the 10x Genomics resources site. PBMC_1 dataset includes Single Cell Multiome ATAC + Gene Expression (https://www.10xgenomics.com/resources/datasets/pbmc-from-a-healthy-donor-granulocytes-removed-through-cell-sorting-10-k-1-standard-2-0-0). PBMC_2 includes Single Cell Gene Expression (https://www.10xgenomics.com/resources/datasets/10k-human-pbmcs-3-ht-v3-1-chromium-x-3-1-high) and Single Cell ATAC (https://www.10xgenomics.com/resources/datasets/10-k-peripheral-blood-mononuclear-cells-pbm-cs-from-a-healthy-donor-1-standard-1-2-0).

Transcriptome data were aligned to hg38 reference genome using STARSolo (Kaminow *et al.* 2021), providing gencode v36 as a gene model (Harrow *et al.* 2012). ATAC data were aligned to hg38 reference genome using bwa (Li, 2013), we then extracted the counts over 5 kb windows spanning the entire genome. For all datasets, we calculated PCA, which was used as input for MOWGAN. Cell clusters were identified using the Planted Partition Block Model (PPBM), implemented in schist.

### 2.6 Embryonic mouse brain datasets

Count matrices for murine Embryonic Brain were downloaded from 10x Genomics resources site. EMB 1 dataset includes Single Cell Multiome ATAC + Gene Expression (https://www.10xgenomics.com/datasets/fresh-embryonic-e-18-mouse-brain-5-k-1- standard-2-0-0). EMB_2 includes Single Cell Gene Expression (https://www.10xgenomics.com/datasets/5-k-mouse-e-18-combined-cortex-hippocampusand- subventricular-zone-nuclei-3-1-standard-6-0-0) and Single Cell ATAC (https://www.10xgenomics.com/datasets/fresh-cortex-hippocampus-and-ventricular-zonefrom- embryonic-mouse-brain-e-18-1-standard-1-2-0).

### 2.7 PBMC scCUT&tag-pro

H3K27me3 and antibody derived tag (ADT) data for PBMC profiled with scCUT&Tag-pro (Zhang *et al.* 2022) were downloaded from https://zenodo.org/record/5504061 (Lab, 2021). R objects were converted to hdf5 and then loaded into scanpy objects. We retained cell type annotation provided with the datasets. Data were normalized and log transformed before computation of the PCA embedding.

### 2.8 Patient-derived colorectal cancer organoids

Samples from three individuals with liver metastatic gastrointestinal cancers were obtained following written informed consent, in line with protocols approved by the San Raffaele Hospital Institutional Review Board and following procedures in accordance with the Declaration of Helsinki of 1975, as revised in 2000. Patient-derived organoid (PDO) cultures were established as previously reported (Bertotti *et al.* 2011, Tedesco *et al.* 2022).

scGET-seq was performed as previously described (Tedesco *et al.* 2022, Cittaro *et al.* 2023) on a Chromium platform (10x Genomics) using ‘Chromium Single Cell ATAC Reagent Kit’ V1 chemistry (manual version CG000168 Rev C) and ‘Nuclei Isolation for Single Cell ATAC Sequencing’ (manual version CG000169 Rev B) protocols. The provided ATAC transposition enzyme (10x Tn5; 10x Genomics) was replaced with a sequential combination of Tn5 and TnH functional transposons in the transposition mix assembly step. Specifically, a transposition mix containing 1.5 μl of 1.39 μM Tn5 was incubated for 30 min at 37°C, then 1.5 μl of 1.39 μM TnH was added for a 1-h incubation. Nuclei suspensions were prepared to get 5000 nuclei as target nuclei recovery. Final libraries were loaded on a Novaseq6000 platform (Illumina) to obtain 100 000 reads per nucleus, and a custom-read 1 primer was added to the standard Illumina mixture (5′-TCGTCGGCAGCGTCTCCGATCT-3′).scRNA-seq was performed on a Chromium platform (10x Genomics) using ‘Chromium Single Cell 3ʹ Reagent Kits v3’ kit manual version CG000183 Rev C (10x Genomics). Final libraries were loaded on a Novaseq6000 platform (Illumina) to obtain 50 000 reads per cell.

### 2.9 Analysis of PBMC datasets

Standard processing was applied to filter and normalize the data: for RNA, cells with <200 expressed genes and genes present in <10 cells were discarded; for ATAC, cells with >30% of captured regions and regions common to >80% of cells were selected. For PBMC_1, we further removed all cells that were not shared across the modalities.

We used PCA as the embedding scheme for all datasets. Cell clusters were identified using a PPBM provided by schist (Morelli *et al.* 2021).

Cell type annotation was defined on RNA PBMC_1 by evaluating cell markers, as illustrated in the Scanpy tutorial (https://scanpy-tutorials.readthedocs.io/en/latest/pbmc3k.html). The annotation was directly transferred to ATAC PBMC_1 by cell identity. Annotations were transferred to PBMC_2 using label transfer function provided by schist.

When we tested the integration of four modalities, known cell types were converted to the ones provided by Zhang *et al.* (2022) according to the following schema:

$$\begin{array}{c} {\left(\text{CD}4\;T\;\text{cells},\;\text{CD}8\;T\;\text{cells}\right)}_{10x},\;\\ {\left(\text{CD}4\;T,\;\text{CD}8\;T,\;\text{Other}\;T\right)}_{\text{scCUT}\&\text{Tag}-\text{pro}}:\;T\;\text{cells}\\ {\left(\text{CD}14+\text{Monocytes},\;\text{FCGR}3A+\text{Monocytes}\right)}_{10x},\;\\ {\left(\text{Mono},\;\text{Other}\right)}_{\text{scCUT}\&\text{Tag}-\text{pro}}:\;\text{Monocytes}\\ {\left(B\;\text{cells}\right)}_{10x},\;{\left(B\right)}_{\text{scCUT}\&\text{Tag}-\text{pro}}:\;B\;\text{cells}\\ {\left(\text{NK cells}\right)}_{10x},\;{\left(\text{NK}\right)}_{\text{scCUT}\&\text{Tag}-\text{pro}}:\;\text{NK cells}\\ {\left(\text{Dendritic cells}\right)}_{10x},\;{\left(\text{DC}\right)}_{\text{scCUT}\&\text{Tag}-\text{pro}}:\;\text{Dendritic cells} \end{array}$$

### 2.10 Analysis of embryonic mouse brain datasets

Standard processing was applied to filter and normalize data: for RNA, cells with <200 expressed genes and genes present in <5 cells were discarded; for ATAC, cells with <500 peaks detected and peaks detected in <5 cells were discarded. PCA was used as an embedding strategy for all datasets. Cell clusters were identified using PPBM provided by schist (Morelli *et al.* 2021).

Cell type annotation was defined on RNA EMB_1, evaluating cell markers. Cell markers for different cell types were retrieved from the Azimuth project (Hao *et al.* 2024), each cell was scored for multiple cell types and the maximal score was retained. To annotate cells at a coarser level, we applied Leiden clustering; for each cell type, we computed the median score for each group, lastly groups were assigned the label with the highest median score.

### 2.11 Analysis of PDOs

Sequencing reads for scGET-seq were processed as previously described (Cittaro *et al.* 2023). Briefly, we counted the number of aligned reads in 5 kb windows spanning the genome, both for tn5 and tnH enzymes. We used raw counts to fit Zero Inflated Poisson distributions at cell level, which were in turn used to estimate the excess of accessible and compact chromatin in each bin. We removed bins with coverage lower than the 90th percentile and cells with <2000 counts. The count matrices were subjected to tensor train decomposition, used as low-dimensionality representation of the data.

Sequencing reads for scRNA-seq were aligned to hg38 reference genome using STARSolo (Kaminow *et al.* 2021), using GENCODE v36 as gene model (Harrow *et al.* 2012). We removed cells that (i) were identified as doublets by scrublet (Wolock *et al.* 2019), (ii) had more than 25% of mitochondrial reads and (iii) having <2000 genes identified. After normalization, identification of highly variable genes, regression of cell cycle and scaling, we obtained low-dimensional representation by PCA.

We used 20 components of tensor train decomposition and PCA as input for MOWGAN with default parameters, computing three different models, one for each organoid, which were then concatenated so that output data have a model label. We used schist to transfer batch identity from input data to MOWGAN output and retained only synthetic data having model label equal to the transferred label.

We used schist (Morelli *et al.* 2021) to infer multimodal cell clusters (*scs.inference.nested_nsbm_multi*) on the integrated dataset. Once cell clusters have been identified, we used the cell marginals (i.e. the probability of a cell to be assigned to any cluster) as lineage specifications in CellRank (Lange *et al.* 2022), so that driver features could be identified in any dataset (e.g. gene drivers for scRNA-seq, region drivers for scGET-seq).

In order to produce a unified UMAP embedding, we first computed separate UMAP for RNA and GET layers, initializing with PAGA positions (Wolf *et al.* 2019) calculated on RNA using level 4 of the Nested Stochastic Block Model (NSBM) hierarchy. We then performed domain adaptation by unbalanced optimal transport based on Sinkhorn algorithm (Chizat *et al.* 2016) implemented in PythonOT (Flamary *et al.* 2021), using level 4 of the NSBM hierarchy as anchors for semisupervised fit.

Transcription factor activity was evaluated on scRNA-seq data, excluding MOWGAN synthetic data), using decoupler (Badia-I-Mompel *et al.* 2022) with default parameters. To calculate the activity of transcription factors in scGET-seq data we first calculated total binding affinity (Molineris *et al.* 2011) of HOCOMOCO v11 motifs (Kulakovskiy *et al.* 2018) in each genomic bin, using bionumpy (Rand 2022). We selected the top 200 region drivers for each cluster, having positive correlation and q-value lower than 1e−20, for a total of 1253 regions. We then calculated the motif score *M* as

$$S=X_{m}\cdot T,\;M=\sqrt{\left|\left(S\right)\right|}\mathrm{sgn}S$$

where *X_m_* is the accessibility value estimated on scGET-seq data and *T* is the total binding affinity (TBA) value for each genomic bin. Once we obtained *M*, we regressed out coverage and scaled the resulting matrix. Colorectal cancer (CRC) Intrinsic Subtypes (CRIS) signatures have been collected from CMSCaller package (Eide *et al.* 2017).

### 2.12 Gene set enrichment analysis

Each dataset was split into two partitions, train and test, in 70:30 proportions, respectively. We applied scanpy’s *sc.tl.rank_genes_groups* (*method* = ‘wilcoxon’, *reference* = ‘rest’, *use_raw* = False) on the test split, retaining features with adjusted *P*-value lower than 5e−3. We sorted features according to decreasing fold-change and selected the top 100 genes in RNA and top 500 regions in ATAC for each annotated cell type. These feature lists were saved as gene sets (or region sets) for GSEA analysis.

We applied rank_genes_groups with the same parameters on the training splits and used the log fold change as ranking metric for preranked GSEA implemented in gseapy (Fang *et al.* 2023). After all cell types had been tested, GSEA results were collected in a confusion matrix reporting the Normalized Enrichment Score (NES) for each pair of cell types. In other words, the confusion matrix reports, for each column, the NES of a given cell type when compared to features selected for all possible cell types.

We divided the predicted values by the column-wise absolute maxima, so that NES are scaled in the [−1, 1] interval. Positive NES values arise when the top-ranking features (e.g. features with the most positive fold changes) are included in the feature set, whereas negative NES value arise when features with the most negative fold changes are included in the feature sets, hence could be interpreted as negative outcomes. Therefore, positive and negative values on the matrix diagonal can be interpreted as true positives (TP) and false negatives (FN), respectively; similarly positive and negative off-diagonal values are interpreted as false positives (FP) and true negatives (TN). Scaled values are summed and used to calculate weighted accuracy as TP + TN/(TP + TN + FP + FN). Since NaN can be generated by gseapy, we substituted them with zeros. Unweighted accuracy can be calculated by taking the sign of each NES instead of its scaled value and it is reported in Table S1.

### 2.13 Other tools

All tools considered for benchmarks were downloaded and used following the respective tutorials. We used the first 10 principal components for each dataset as input for Pamona, SCOT and scMMGAN, whereas COBOLT was run using normalized count matrices, as required.

The tools were tested with the following parameters:

$$\begin{array}{c} \text{Pamona}:\;n\_\text{neighbors}=40,\;\\ \text{Lambda}=10,\;\text{output}\_\text{dim}=10\\ \text{SCOT}:\;k=50,\;e=0.0005,\;\text{normalize}=\text{True}\\ \text{scMMGAN}:\;\text{training}\_\text{steps}=20000,\;\\ \text{batch}\_\text{size}=64,\;\text{nfilt}=128,\;\\ \text{learning}\_\text{rate}=0.0001,\;\text{lambda}\_\text{cycle}=1,\;\\ \text{lambda}\_\text{correspondence}=1,\;\\ \text{add}\_\text{noise}=\text{False},\;\text{use}\_\text{bn}=\text{True}\\ \text{COBOLT}:\text{lr}=0.005,\;n\_\text{latent}=10,\;\text{num}\_\text{epochs}=100. \end{array}$$

## 3 Results

### 3.1 Overview of MOWGAN

The core component of the framework is a WGAN with gradient penalty (WGAN-GP). A WGAN-GP is a generative adversarial network that uses the Wasserstein (or Earth-Mover) loss function and a gradient penalty to achieve Lipschitz continuity (Arjovsky *et al.* 2017, Gulrajani *et al.* 2017). Like any other GAN, the WGAN-GP is composed of two subnetworks, called *generator* and *critic*. MOWGAN’s *generator* outputs a synthetic dataset where cell pairing is introduced across multiple modalities.

**Figure 1.**
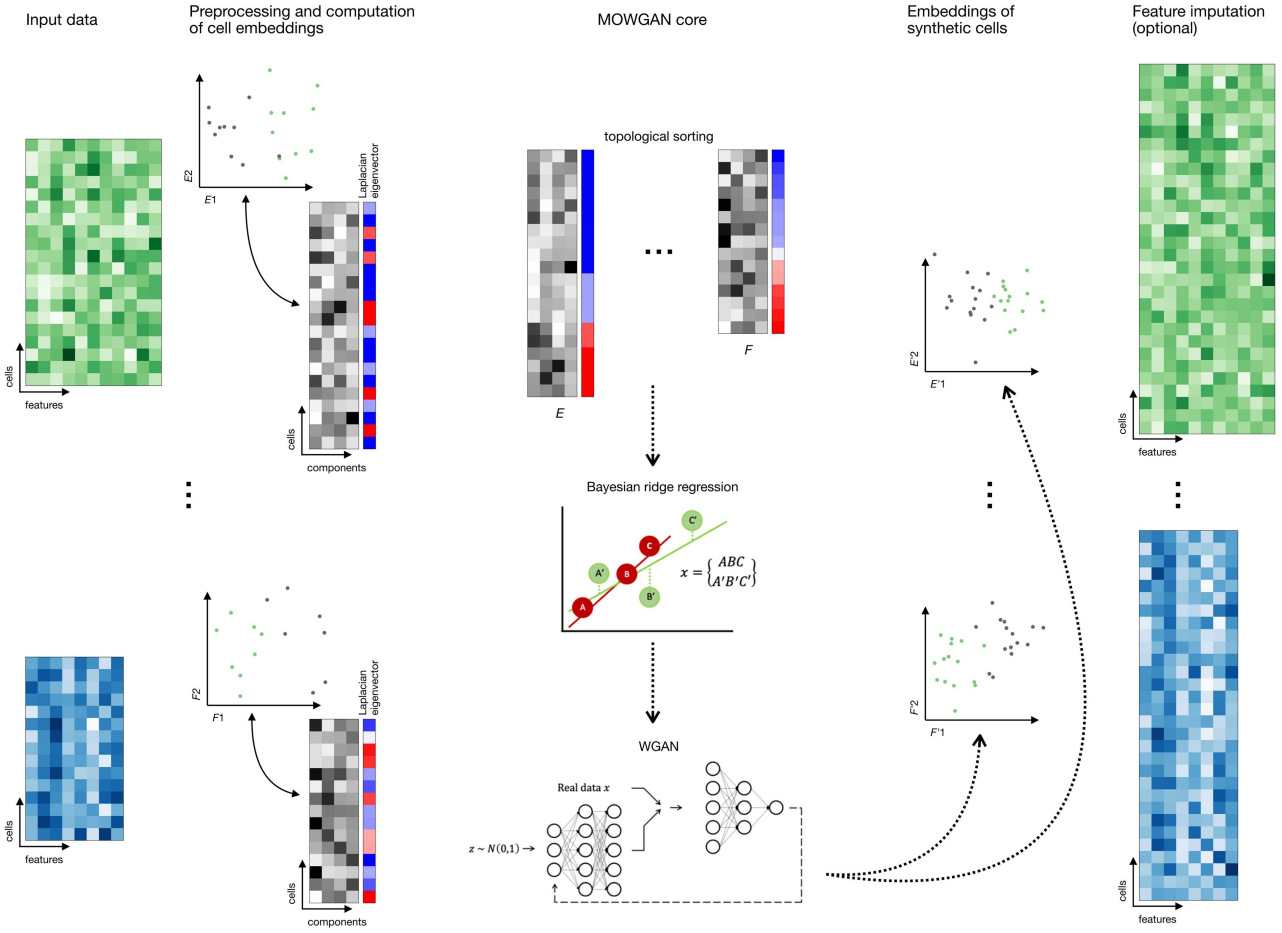
Overview of MOWGAN. MOWGAN is a framework for the generation of synthetic multi-omics data. Given unpaired single-cell data (e.g. scRNA-seq and scATAC-seq from separate assays), MOWGAN learns both the specificity and the relationship between modalities, returning a new, paired, dataset. Input data are processed according to relevant pipelines to generate cell embeddings that are given as input to MOWGAN. First, Laplacian Eigenmap is calculated to sort cell embeddings according to their structural features. During mini-batch training, every mini-batch is used to fit a Bayesian ridge regressor used to score the most similar mini-batch from the other modality. Once mini-batches are formed, they are used to train a WGAN-GP. The final output is a single-cell multi-omics dataset including embeddings generated by the WGAN-GP. Optionally, feature values for the newly generated data are reconstructed using kNN-regression.

MOWGAN’s inputs are molecular layers embedded into a feature space having the same dimensionality *C* (Fig. 1). In principle any dimensionality reduction technique could be used (e.g. PCA, SVD, etc.). As WGAN-GP training is performed in mini-batches, iteratively sampling random subsets of cells, it would be favorable that (1) each mini-batch contains cells of the same types and that (2) cells in any mini-batch from one modality is presented to the set of most similar cells in the mini-batch from the other modalities. To match the first condition and capture local topology, MOWGAN sorts cells in each embedding according to the first component of their Laplacian Eigenmaps (LE), these calculated on each modality. This step is justified by the fact the eigenvectors of the graph Laplacian, ordered by increasing eigenvalue, grasp the local data structure in terms of cell-to-cell distance (Belkin et al. 2002); therefore, we increase the chance that similar cells are chosen together when mini-batches are sampled from the embedding matrix. To match the second condition, once a mini-batch is selected from one modality (usually RNA), we fit its embedding to its LE values using a Bayesian ridge regressor *R*; next, *n* mini-batches (default *n *=* *50) are selected from the other modality and *R* is applied on their embeddings and LE values to produce a score used to select the mini-batch with local properties more like the first, increasing the chance to pair samples drawn from same cell types in both modalities. This procedure is repeated for all given modalities, always using one as a reference (e.g. RNA). In preliminary implementations of MOWGAN, without this ‘pre-match’ step of LE sorting and regression, we noticed the WGAN-GP could only capture global topology of the data, resulting in random mixing of cell types (Supplementary Fig. S1).

The mini-batches are combined to form a tensor (*N*, *M*, *C*), where *N* is the number of cells in a mini-batch (default *N *=* *256), *M* is the number of modalities evaluated (2 in case of just the RNA and ATAC layers), and *C* is the rank of the input embeddings. In any case, *C* is set at most to the lowest matrix rank across all modalities.

Semisupervised training is implemented to include prior information about the dataset. This allows to model, for example, batch-specific models that are eventually merged. Lastly, MOWGAN can explicitly include paired data during training, skipping the pre-match step.

We implemented MOWGAN in Python using Tensorflow libraries to support of GPU-enabled hardware and scale to large datasets.

### 3.2 MOWGAN preserves data topology and biological information

We prototyped MOWGAN on public scRNA and scATAC data of PBMC and embryonic mouse brain (EMB) for which paired and unpaired experiments were available (10x Genomics datasets). The paired datasets (PBMC_1 and EMB_1) are true multimodal experiments, with transcriptome and epigenome data from the same cells; the unpaired datasets (PBMC_2 and EMB_2) include data from two separate experiments, one for each modality.

We evaluated MOWGAN output in terms of shared information between layers [adjusted mutual information of cell clusters (AMI)], integrability [Local Inverse Simpson’s Index (LISI)] (Korsunsky *et al.* 2019) and ability to recapitulate biological information within the same modality. Multimodal data can be leveraged as ‘molecular bridges’ to map information from one modality to another (Hao *et al.* 2024), exploiting the cell identities. We performed experiments in which either the true multimodal data or synthetic cells generated by MOWGAN were used as data bridges to transfer labels from RNA to ATAC modality.

**Figure 2.**
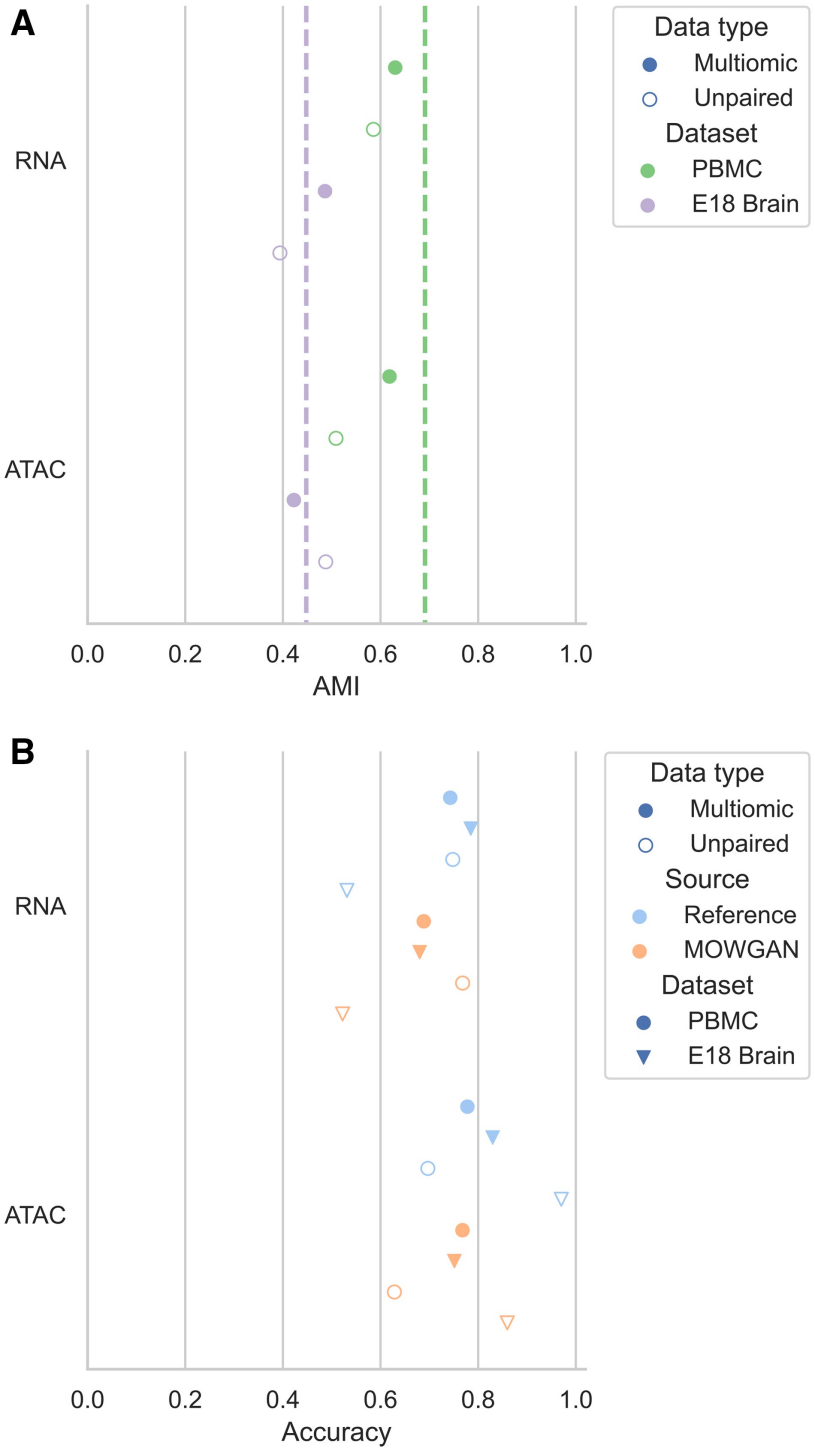
Evaluation of the ability to induce cell pairings. (A) Graph showing the AMI between cell clusters in each modality and the clusters transferred from the other modality using MOWGAN. The dashed vertical lines indicate the baseline AMI computed transferring labels using cell identities on paired data. (B) Graph reporting the accuracy values for cell type predictions in MOWGAN outputs. In all settings, MOWGAN was trained using 70% of cells in each dataset, whereas 30% was used to generate feature marker sets for GSEA analysis. Reference values were obtained by analyzing the training data before MOWGAN was run.

First, we identified a set of cell clusters *T* (‘truth’) in each modality using a PPBM (Zhang and Peixoto 2020). We denote the set of transferred cell clusters using data bridges as *P* (‘prediction’). Labels are propagated from the first modality *D*_1_ to MOWGAN and then from MOWGAN to the second modality *D*_2_ using label transfer function provided by schist package (Morelli *et al.* 2021). As every cell in *D*_1_ and *D*_2_ will be annotated by *T* and *P*, we could calculate mutual information. When measured on multimodal data where bridge is cell identity, mutual information was computed comparing *T* annotations of RNA and ATAC and found below 1 in both PBMC and EMB (AMI_PBMC_ = 0.691, AMI_EMB_ = 0.448; Fig. 2A, Supplementary Table S1), indicating that different modalities encode slightly different cell properties.

When cell clusters were transferred using MOWGAN, we observed slightly lower values (Fig. 2A, Supplementary Table S1), as expected. Compared to their paired counterparts, PBMC_2 and EMB_2 showed lower AMI values, which could be also expected, as both are composed of two separate experiments which were acquired, analyzed, and released over a long extent. Of note, reference AMI values for unpaired data cannot be computed as cell identities are not conserved across modalities.

Data integrability was measured using LISI, a measure of the amount of data mixing in the cell neighborhood. If two datasets (*A, B*) are well integrated, we may expect the neighbors of every cell in *A* to be well represented in *A* and *B* datasets, and *vice versa*. When integration fails, most of the neighbors of every cell in *A* (*B*) will be from dataset *A* (*B*) only. LISI is calculated within the same modality (*D*_1_ or *D*_2_ with MOWGAN) and it should be intended as a global measure of similarity of two datasets. It varies from 1 (perfect separation) to the number of modalities *M* (perfect mixing).

**Figure 3.**
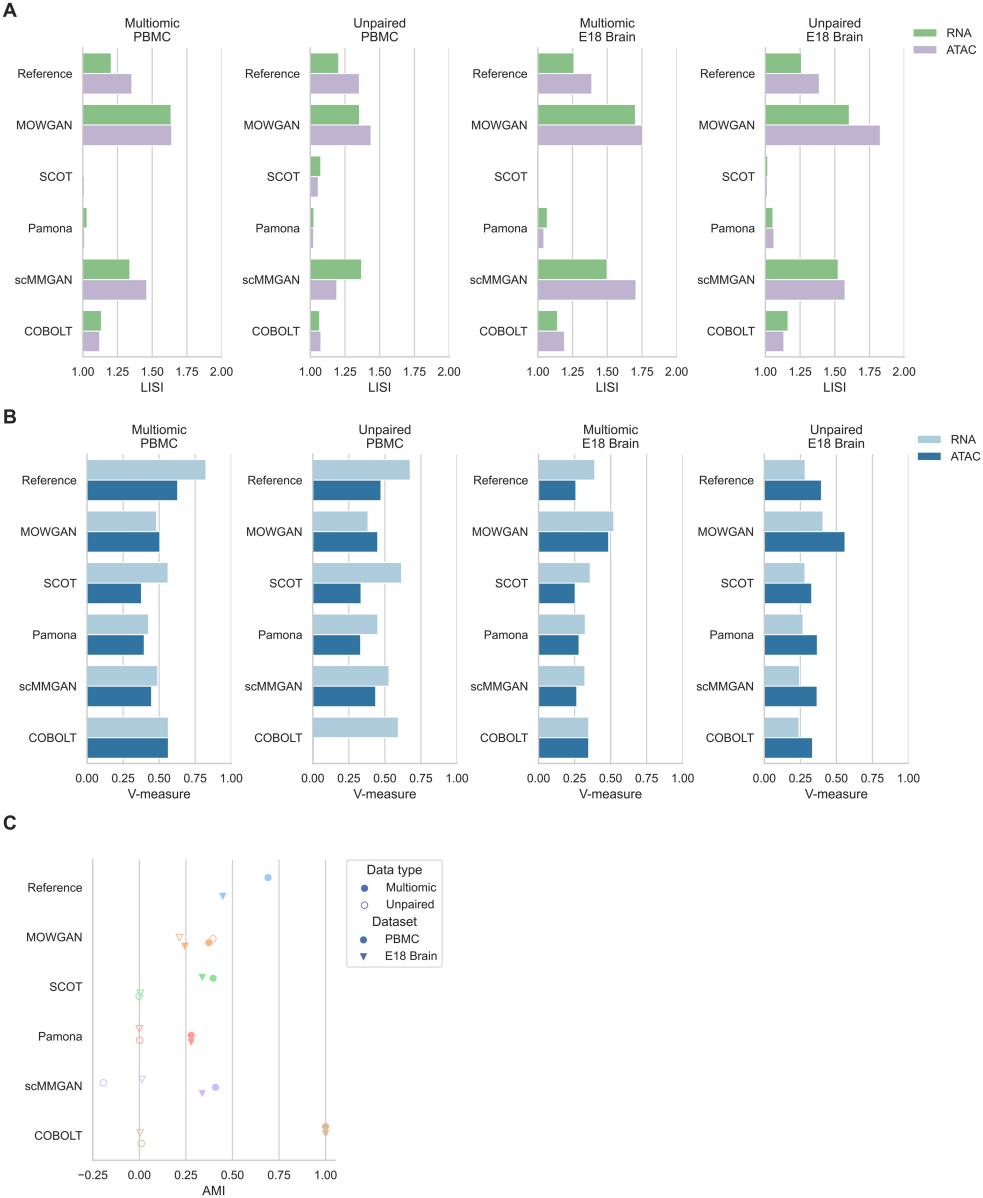
Comparison with other tools for multimodal single-cell integration. (A) Histograms showing LISI scores in four datasets for two different modalities. LISI values for SCOT and Pamona have no meaningful interpretation, due to the fact both generate a new embedding in a coordinate system different from the respective input. (B) Histograms showing V-measure scores were obtained comparing annotated cell types and cell clusters identified in each modality in four datasets. (C) Graph showing AMI values between cell clusters identified in each datasets and cell clusters after each tool is applied. The highest values detected for COBOLT in paired experiments are due to COBOLT generating only one embedding for multimodal data.

Baseline measures of integrability were dependent on the modality and similar in both biological contexts (Fig. 3A, Supplementary Table S2). Data generated by MOWGAN had higher LISI values (Fig. 3A, Supplementary Table S2), indicating that it is able to compute synthetic data that resemble the input.

Lastly, we evaluated the ability to transfer biological information as the ability to correctly identify cell types. To this end, we randomly partitioned each dataset in two splits, train and test, with 70:30 proportion. For each cell type, we identified the set of marker features in the test subset as the top significant features ordered by descending fold change. We then processed the train subsets with MOWGAN, inducing pairings between RNA and ATAC, and calculated the feature-wise fold change in every cell type. Since the WGAN-GP itself returns the embeddings for synthetic cells but not feature values, we used kNN-regression to impute them (see Section 2).

Fold changes were then used to perform a preranked GSEA (Subramanian *et al.* 2005) against all marker sets. The endpoint of such GSEA analysis is a confusion matrix with NES values for every pair of true and predicted cell types, one confusion matrix for each dataset. Reference values were computed directly on the training set (i.e. on the true data).

Performance was evaluated in terms of accuracy: NES values were scaled to the column-wise absolute maxima (i.e. NES are scaled to the maximum among predictions for each cell type); positive and negative values on the diagonal were summed to quantify the TP and FN, respectively. Off-diagonal positive and negative values quantified FP and TN, respectively. Accuracy of MOWGAN was in line with reference values for all experiments (Fig. 2B) with high correlation (*r* = 0.927, *P* = 9.278e−4), indicating that lower accuracies are due to intrinsic dataset properties and do not arise from biases induced by MOWGAN.

In all, these results indicate that MOWGAN can induce appropriate pairings in synthetic data which, in turn, can be used to perform bridge integration of multiple modalities.

### 3.3 Comparison with other tools

We benchmarked MOWGAN against four tools that share similar design principles: Pamona (Cao *et al.* 2021), SCOT (Demetci *et al.* 2022b), COBOLT (Gong *et al.* 2021) and scMMGAN (Amodio *et al.* 2022). Pamona and SCOT use optimal transport theory to project each modality on a single latent embedding. The new embeddings can be processed by standard workflow to identify cell clusters. scMMGAN projects data of each modality to the embedding defined on the other modality, so that two modalities can be aligned in a single space. Similarly to Pamona and SCOT, the resulting embedding can be used to identify cell clusters. COBOLT, instead, projects unpaired data on the embedding obtained from paired data, to perform bridge integration.

For each tool, we evaluated the consistency between cell types and cell clusters derived from the generated embeddings in all datasets. Cell types were annotated once in both PBMC and both EMB datasets, to be consistent across all experiments. In addition to AMI between *P* and *T* cell clusters, we measured completeness (i.e. all cells of any cell type are clustered together), Homogeneity (i.e. all cells of any cluster belong to the same cell type), and their harmonic mean (V-measure). In general, we observed better performances in multimodal data compared to unpaired ones and on the RNA layer compared to the ATAC layer (Fig. 3B, Supplementary Table S2). Moreover, we observed higher performances in PBMC compared to EMB, possibly due to highest complexity of neuronal tissues compared to blood composition.

While all tools performed in comparable ways, MOWGAN displayed a consistent behavior across all datasets, whereas other tools were penalized in EMB. MOWGAN also performed similar to scMMGAN, with which shares some design principles as both rely on deep generative models. Of note, COBOLT failed in integrating ATAC data in PBMC_2 (V_PBMC_2_ = 0.009) and its highest performance in multimodal data (PBMC_1 and EMB_1) was due to the fact it explicitly uses cell identities to integrate two modalities, projected in a single space.

We noticed some tools induce spurious cell type associations (Supplementary Fig. S2). For example, in the PBMC experiment, SCOT switched B cells with NK/CD8 T cells in PBMC_1 and Monocytes with T cells in PMBC_2, scMMGAN suffers of several cell type switches in all the tested datasets, COBOLT completely shuffled cell types in the ATAC layer in PBMC_2.

Integrability measured by LISI showed comparable performance of MOWGAN and scMMGAN (Fig. 3A), again possibly due to the fact both are based on GAN. We observed low LISI values for SCOT and Pamona; however, they should not be considered in the comparison as both tools generate embeddings in a new reference system that is necessarily different from the input one.

Lastly, we compared the ability of each tool to preserve data structure. To this end, we identified cell clusters using PPBM in the original and in the processed data (i.e. in the synthetic cells generated by MOWGAN or in the cell projections in computed spaces for other tools), then we computed the mutual information. We found all tools share similar performances on multimodal dataset (Fig. 3C), but MOWGAN was the only tool sustaining comparable performance on unpaired data, while the remaining tools were characterized by AMI values close to 0 (Fig. 3C, Supplementary Table S2). In case of paired data, as for PBMC_1 and EMB_1, there is the possibility to train the WGAN-GP so that mini-batches explicitly include paired cells, skipping the LE-sort and ridge regression steps. When MOWGAN ran in this configuration, we observed a sensible increase of AMI values (AMI_PBMC_1_ = 0.538, AMI_EMB_1_ = 0.455), suggesting that experimental pairings should be used whenever possible. Nevertheless, we decided not to discuss further such configuration to allow for fair comparisons.

In all, these results indicate that all tools but COBOLT preserve the local structure of the input data as clustering properties are conserved in the data transformations. In this regard, no tool outperforms the others, suggesting an upper bound for integration tasks. However, most tools, except for MOWGAN, did not guarantee the correct cell type associations, a further evidence of the ability of MOWGAN to generate good quality and meaningful data pairings.

We recorded running times in order to perform the comparisons (Supplementary Fig. S3, Table S3), MOWGAN runs in <3 hours on all datasets and lies in between the fastest and the slowest tool (scMMGAN and SCOT, respectively). It should be added that MOWGAN, as scMMGAN, is bound to execute on GPU-accelerated hardware.

### 3.4 MOWGAN extends the integration to high number of modalities

The major advantage of MOWGAN is that it can integrate more than two molecular layers without modifying the model architecture. To test this feature, we took advantage of the availability of different single-cell profiles for PBMC; in particular, we attempted the integration of PBMC_1 dataset with H3K27me3 and ADT data obtained with scCUT&TAG-pro (Zhang *et al.* 2022) on PBMC, to achieve a four-layer integration. It is worth underline that the pairings between ADT and H3K27me3 and between RNA and ATAC were not exploited in this experiment. We observed high integrability between original and synthetic data generated with MOWGAN, with the lowest performance detected for H3K27me3 (Supplementary Table S4).

**Figure 4.**
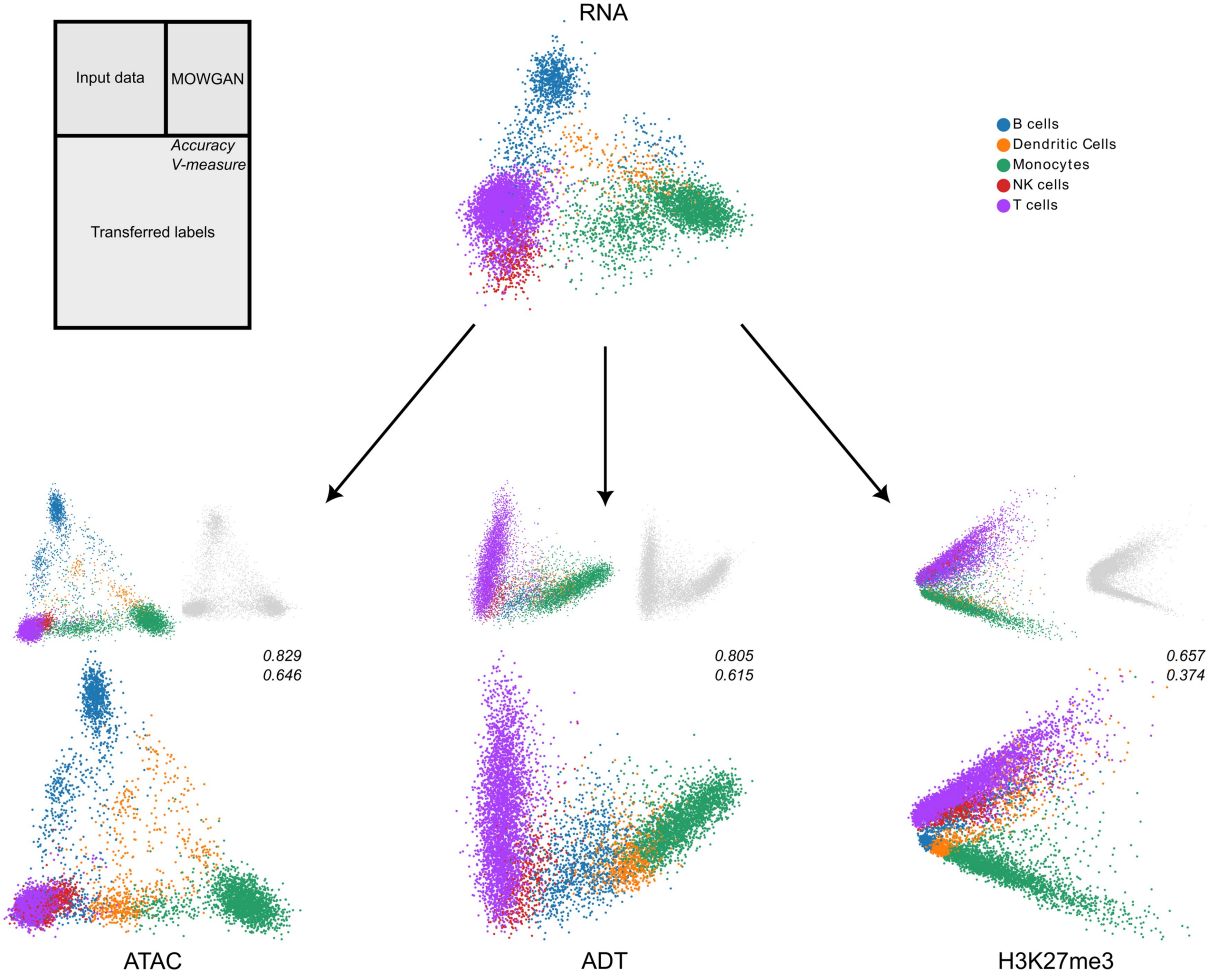
Integration of multiple single-cell modalities. We report the low-dimensional embeddings (PCA) of multiple single-cell profiles of PBMC. After data integration, we used MOWGAN to bridge cell type annotations from RNA to ATAC, ADT, and H3K27me3 layers. For each modality receiving the annotation, we report three embeddings: the input data colored by cell type (top left), the embedding of data generated by MOWGAN (top right), and the input data colored by the trans-ferred cell type (bottom). We report values of accuracy and V-measure for each transfer as well.

We used MOWGAN to bridge cell type annotations from RNA to all the remaining modalities, then compared with the given cell type (Fig. 4). We found similar results for ATAC and ADT, both in terms of accuracy (>0.8) and V-measure (>0.6), whereas H3K27me3 performed worst, suggesting RNA, ATAC, and ADT share similar resolution power with respect to cell types. On the other hand, H3K27me3 clearly separated myeloid and lymphoid lineages with lower resolution power in the latter. An analogous behavior of H3K27me3 has been previously described (Wu *et al.* 2021).

Of note, we found the performance of inverse labeling, from any modality to RNA, equally high, if not better (Supplementary Table S4). In all, these results show how MOWGAN can effectively compute synthetic pairings for multiple modalities, overcoming a limitation that is common to many available tools.

### 3.5 Semisupervised training improves results and unveils hidden cell populations in colorectal cancer organoids

An ideal tool to integrate unpaired data would be able to work in unsupervised manner, that is, it would learn the whole data structure from the data themselves. That requires the algorithm to model several confounders including technical noise due to different technologies (Mereu *et al.* 2020, Zhu et al. 2020), biological noise (Paszek 2014, Eling *et al.* 2019) and batch effects (Tran *et al.* 2020). For this reason, we tested the ability of MOWGAN to work with multiple samples in semisupervised way specifying the sample identity in the training step.

We profiled three PDO of liver metastatic CRC by scRNA-seq and scGET-seq (Tedesco *et al.* 2022) (Supplementary Table S5). Cells for each PDO were split in two aliquots before single-cell preparation, so that data are paired at sample level (i.e., single cells represent the same populations) but not at modality level. Analysis of copy number alterations at single-cell level revealed that all PDOs are genetically uniform and essentially monoclonal (Supplementary Fig. S4A). These findings were previously confirmed for CRC6 and CRC17 by bulk exome sequencing (Tedesco *et al.* 2022).

To verify the effectiveness of semisupervised training, we trained one MOWGAN model without specifying the organoid of origin (NB) and a model that instead included such information (B). In practice, to obtain model B, we trained three separated models, one per organoid, sampling batches from the whole dataset, and eventually concatenating the outputs.

We checked the average concordance between the actual organoid labels and the one we obtained transferring it from scRNA-seq to scGET-seq and *vice versa* using MOWGAN as bridge. Model NB showed the lowest performance in terms of accuracy (ACC_RNA_ = 0.111, ACC_GET_ = 0.353) compared to model B (ACC_RNA_ = 0.982, ACC_GET_ = 0.668).

Synthetic data were generally integrable with the original ones, with minor differences between model B (LISI_RNA_ = 1.55, LISI_GET_ = 1.33) and NB (LISI_RNA_ = 1.42, LISI_GET_ = 1.25), in line with previous observations on PBMC and EMB datasets. In all, we conclude that semisupervised learning improves the quality of the generated data and their usability for the integration.

**Figure 5.**
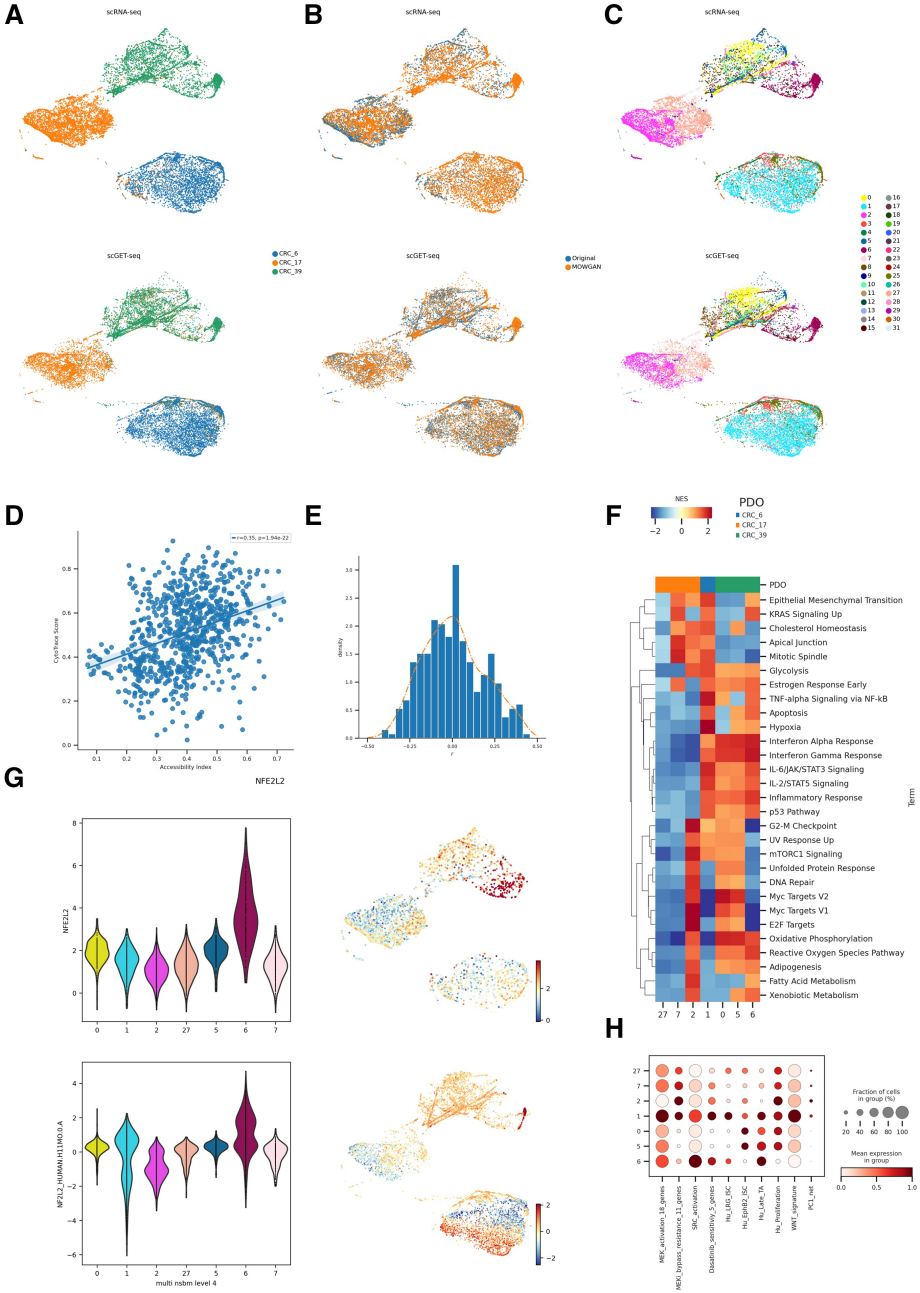
Analysis of CRC PDOs. (A) Unified UMAP embeddings of the scRNA-seq (top) and scGET-seq data (bottom) where single cells have been colored by PDO of origin. (B) Unified UMAP embedding as in (A), cells have been colored according to their data source, whether it is original input data or synthetic data generated by MOWGAN. (C) Same embedding as in A and B, cells have been colored by the level 4 of the NSBM hierarchy inferred on multimodal data. (D) Scatterplot representing the correlation between Chromatin Accessibility Score, calculated on scGET-seq data, and CytoTrace score, calculated on scRNA-seq data. Each point represents the median value of a score in cell groups at level 0 of the NSBM hierarchy. (E) Distribution of the correlation values between Transcription Factor activities, evaluated on scRNA-seq data, and Total Binding Affinity, evaluated on scGET-seq data. The orange line traces the smoothed profile of the histogram (F) Gene Set Enrichment Analysis of most represented cell groups after integration. The heatmap displays the Net Enrichment Score of preranked GSEA, using Hallmark gene sets. (G) NFE2L2 profiles in integrated data. We report the distribution of TF activity and TBA score of NFE2L2 in scRNA-seq (top) and scGET-seq (bottom) for the most represented cell groups. Next to each violin plot we report the relative unified UMAP embedding, colored by the same score. (H) Profile of gene signatures described for drug resistance phenotypes in CRC. The dotplot reports the score of each signature in the most represented cell groups found in integrated data.

Having set the model B is better, we performed further analysis to unveil the biological features of PDOs. We identified cell states using the multimodal capabilities of schist (Morelli *et al.* 2021), which relies on a Stochastic Block Model with overlap. Two kNN graphs, including MOWGAN generated cells, are computed for each modality and merged specifying an edge label depending on the modality, then a NSBM with edge covariates is inferred. Of note, MOWGAN output is represented in the graph as a set of nodes having both types of edges, hence providing the link between modalities. The resulting groups are consistent across modalities and can be used to analyze phenotypes from the transcriptomic and the epigenetic standpoint. We computed a unified embedding using optimal transport framework to visualize data annotations in consistent way (Fig. 5A–C).

Since the rate of transcription depends, among other factors, on genome accessibility, we calculated the CytoTrace score (Gulati *et al.* 2020) from scRNA-seq and a chromatin accessibility score (de Pretis and Cittaro 2022) from scGET-seq. The correlation between the two scores, averaged on the deepest level of NSBM hierarchy, was positive (Fig. 5D, *r *=* *0.35, *P *=* *1.94e−22). In addition, we found a positive correlation between accessibility and CRIS-B signature for CRC (Supplementary Fig. S4B, *r *=* *0.18, *P *=* *4.054e–7). We expected positive correlations in case of appropriate pairing, since CRIS-B subtype has been described associated to EMT phenotype (Isella *et al.* 2017), as well as higher CytoTrace scores and chromatin accessibility (Tam and Weinberg 2013, Arase *et al.* 2017) from scGET-seq.

We then evaluated the activity of various transcription factors from gene expression data, using decoupler (Badia-I-Mompel *et al.* 2022) and a cell-level measure derived from TBA (Molineris *et al.* 2011), computed from chromatin accessibility (see Section 2). Correlation coefficients of the two measures were skewed to positive values (Fig. 5E, skeweness = 0.251), again supporting the good quality of the integration. Among other transcription factors (Supplementary Table S6), we found high correlation for NFE2L2 (*r *=* *0.39, *P *=* *1.742e-28), which regulates stress response (Sanchez-Vega *et al.* 2018) and has been recently found involved in CRC resistance to therapy (O’Cathail *et al.* 2021). Interestingly, NFE2L2 is strongly associated with only one subpopulation in CRC39 (Fig. 5G), indicating the PDOs, while being genetically uniform, display phenotypically diverse cell states.

We focused our attention on level 4 of the NSBM hierarchy as it provided a good trade-off between modularity and detail on the subpopulations within each PDO. We identified 32 clusters, seven of which include >200 cells in both modalities. All PDOs, apart from CRC6, were composed of different subpopulations with distinct phenotypes (Fig. 5F). We scored multiple expression signatures studied for their relevance in drug resistance (Yang *et al.* 2022) and confirmed that both CRC17 and CRC39 included subpopulations that could support a differential response to treatments (Fig. 5H, Supplementary Fig. S4C).

Importantly, these cell populations could be hardly identified by using scRNA-seq or scGET-seq data alone. First, we noticed that the NSBM hierarchy of cell clusters identified in single assays did not strictly follow the PDO identities and, in fact, some clusters are shared across different organoids, particularly in the scGET-seq data. Cell groups identified on the integrated data appear to be more consistent with the organoid identity than groups identified on the single modalities (Supplementary Fig. S4D); this behavior is true at any level of the NSBM hierarchies. Consequently, some phenotypes we observed above could not be clearly assigned to specific subpopulations.

In all, these data confirm that MOWGAN is able to produce good-quality pairings to perform bridge integration across modalities. Moreover, we show that semisupervised training is an effective approach to operate when multiple samples/batches are available.

Methods to probe molecular phenotypes of single cells at high throughput have been available for more than a decade now. Such methods have been rapidly adopted and are now standard techniques in many fields of biological studies. The largest part relies on the analysis of gene expression, for which many technologies are available (Chen *et al.* 2018). Methods to investigate single-cell epigenomes have also increased in number overtaking scRNA-seq (Preissl *et al.* 2023). The development of unimodal approaches, including methods to investigate genome sequences (Gawad *et al.* 2016) or proteomes (Labib and Kelley 2020, Brunner *et al.* 2022), is paralleled by the introduction of multimodal techniques, whose number has also exploded in recent years (Ogbeide *et al.* 2022, Baysoy *et al.* 2023, Vandereyken *et al.* 2023). Unless innovative chemistry/microfluidic strategies are introduced, multimodal investigation will be inherently limited to methods that preserve cell or molecule integrity. Moreover, despite the availability of multimodal techniques, their analysis remains challenging (Lance *et al.* 2022); moreover, they may be less informative of single-assay methods (Booeshaghi *et al.* 2023). For all these reasons, the development of computational strategies to pair unimodal assays is flourishing.

Along this path, we introduced MOWGAN, a DL-based framework that makes use of WGAN to generate synthetic multimodal data for bridge integration of different single-cell assays. Given the heterogeneity of available technologies, we designed MOWGAN to work without the need of ‘biologically principled anchors’ across modalities, such as Gene Activity Score for scATAC-seq data (Granja *et al.* 2021) or Chromatin Silencing Score for repressive histone marks profiled in scCUT&Tag (Wu *et al.* 2021).

Comparisons with other tools on the integration of two unpaired modalities (scRNA-seq and scATAC-seq) show MOWGAN performs like the top-level methods on paired data, suggesting an intrinsic upper bound for this task, but MOWGAN is the only tool able to effectively integrate unpaired data. The major improvement of MOWGAN over existing tools is the possibility of working with more than two modalities. To do so, it aligns all input data to one base modality of choice, typically the transcriptome. Consequently, MOWGAN does not generate a data barycenter, nor it computes an embedding in a latent space. In this regard, MOWGAN could be used in a modular workflow, allowing the adoption of the method of choice to bridge information. We are aware that other tools can operate on a high number of modalities; however, they require partial overlaps among data (Ghazanfar *et al.* 2023) or explicit code refactoring (Amodio *et al.* 2022).

Another important feature of MOWGAN is the possibility to specify experimental pairings, being those cell or sample identities. Semisupervised training of MOWGAN, when possible, reduces the possibility of hallucination, a well-known issue that affects deep generative models (Ji *et al.* 2022), and allows the computation of good-quality and biologically meaningful data.

While it is established that ‘no method works best for all’, we believe that MOWGAN has sufficient power to generalize over various technologies. We showed that the integration of multiple modalities, notwithstanding that data are generated from separate experiments, provides information in a synergistic way, hence revealing hidden patterns. Our findings are particularly relevant in the context of intratumor heterogeneity. In our PDO dataset, all but one sample has been derived from tumors previously exposed to chemotherapy, therefore it is expected they contain (epigenetic) subclones selected for drug resistance; CRC39, on the other hand, has been derived from a naive tumor and it would be tempting to speculate on the existence of subclones conferring drug resistance that could eventually expand upon therapy.

## Supplementary Material

btae300_Supplementary_Data

## Data Availability

scRNA-seq data for three PDOs have been deposited to ArrayExpress with the following ID: EMTAB- 13123. scGET-seq data for CRC39 have been deposited to ArrayExpress with the following ID: E-MTAB-13126. scGET-seq data for CRC6 and CRC17 were downloaded from ArrayExpress (E-MTAB-10219).
