# Supplementary material for: Scalable integration of multiomic single-cell data using generative adversarial networks
Source: Bioinformatics. 2024 May 2;40(5):btae300. doi: 10.1093/bioinformatics/btae300 (PMC11654621; doi:10.1093/bioinformatics/btae300)
Supplement: btae300_Supplementary_Data [file btae300_supplementary_data.pdf]

Scalable integration of multiomic single cell data using generative adversarial networks

Valentina Giansanti[1,2], Francesca Giannese[2], Oronza A. Botrugno[3,4], Giorgia Gandolfi[2], Chiara Balestrieri[2,6], Marco Antoniotti[1,5,7], Giovanni Tonon[2,3,4] and Davide Cittaro[2*]

[1]Department of Informatics, Systems and Communication, Università degli Studi di Milano-Bicocca, Milan, Italy
[2]Center for Omics Sciences, IRCCS San Raffaele Scientific Institute, Milan, Italy
[3]Functional Genomics of Cancer Unit, IRCCS San Raffaele Scientific Institute, Milan, Italy
[4]Vita-Salute San Raffaele University, Milan, Italy
[5]Bicocca Bioinformatics Biostatistics and Bioimaging Centre -- B4, Università degli Studi di Milano-Bicocca, Milan, Italy
[6]Experimental Hematology Unit, IRCCS San Raffaele Scientific Institute, Milan, Italy.
[7]Istituto di Bioimmagini e Fisiologia Molecolare, Consiglio Nazionale delle Ricerche (CNR), Milan, Italy

Supplementary Figures

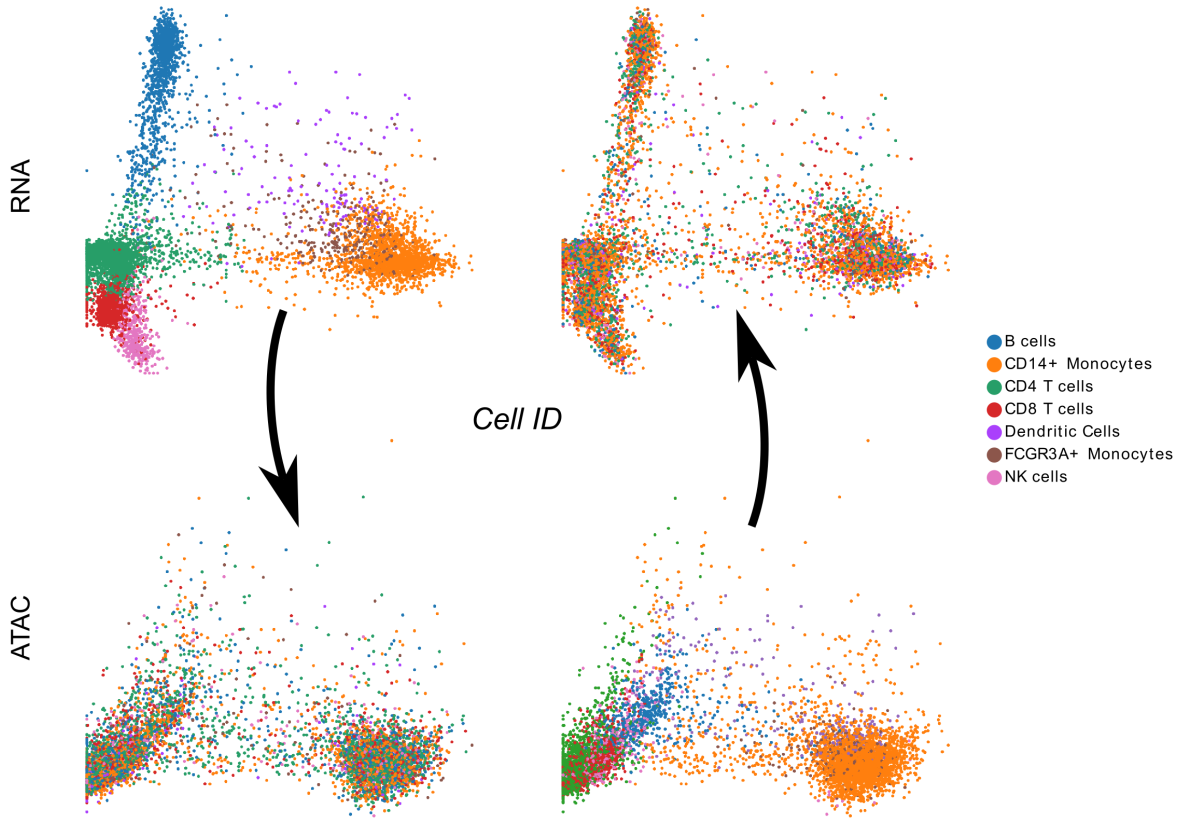

Fig. S1 Low dimensional embeddings (PCA) of PBMC_1 data generated by WGAN-GP trained with random sampling. The overall distribution of points indicates that the WGAN-GP learns data topology correctly. However, when cell types are transferred across modalities (from RNA to ATAC and vice versa) the cell group information is lost.

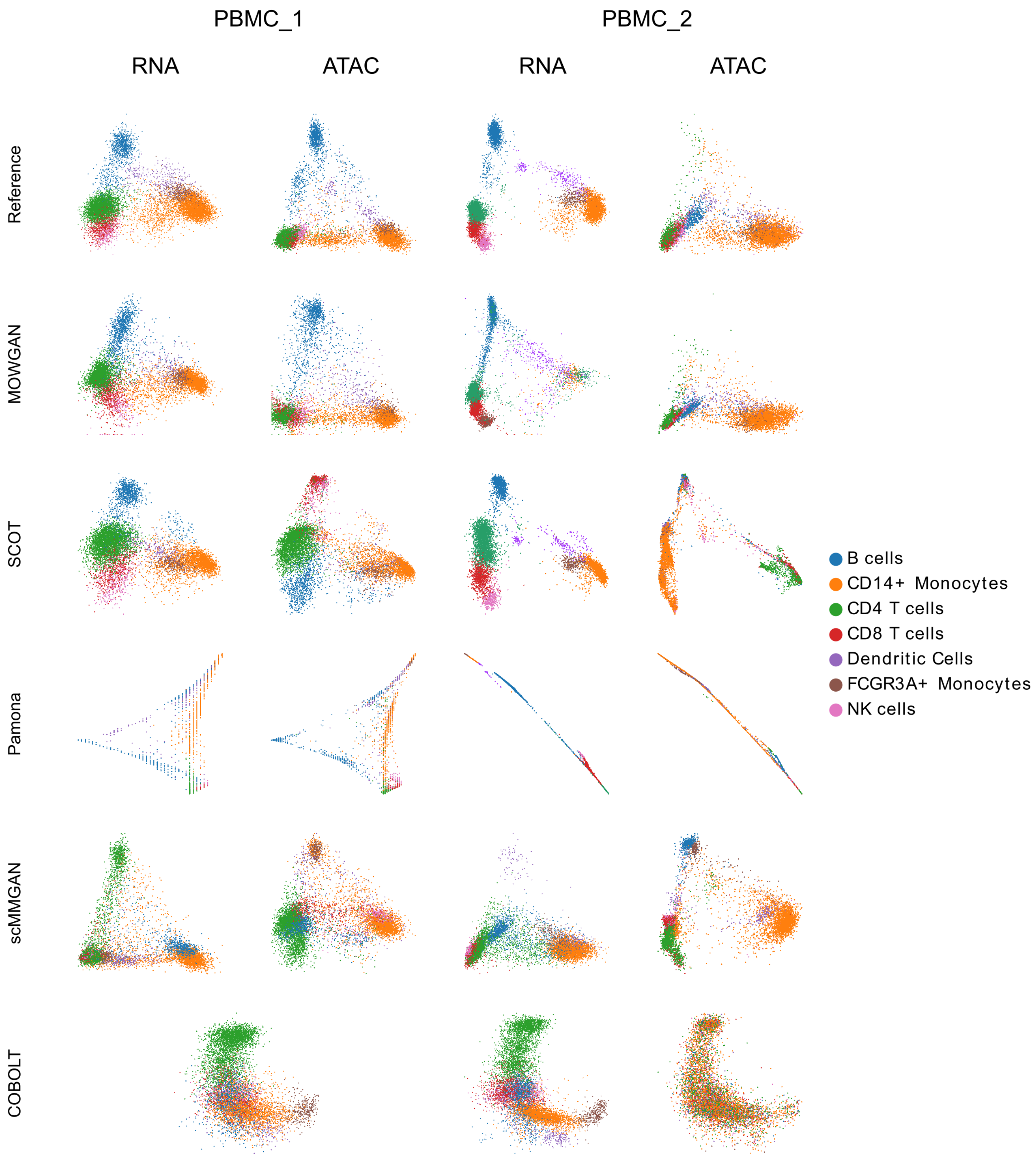

Fig. S2 Low dimensional representation of PBMC_1 and PBMC_2 datasets generated by different tools used for benchmarking. Each row reports the embedding produced by a tool, colored by cell type. As described in the text, the analysis of these embeddings reveals spurious cell type transfers across modalities for some tools (i.e., SCOT, scMMGAN and COBOLT). We report one embedding for PBMC_1 (paired data) produced by COBOLT since it projects both modalities into a single embedding. Embeddings produced by scMMGAN appear inverted compared to the reference as it projects cells of one modality (e.g., RNA) to the embedding learned from the other modality (e.g., ATAC) and vice versa.

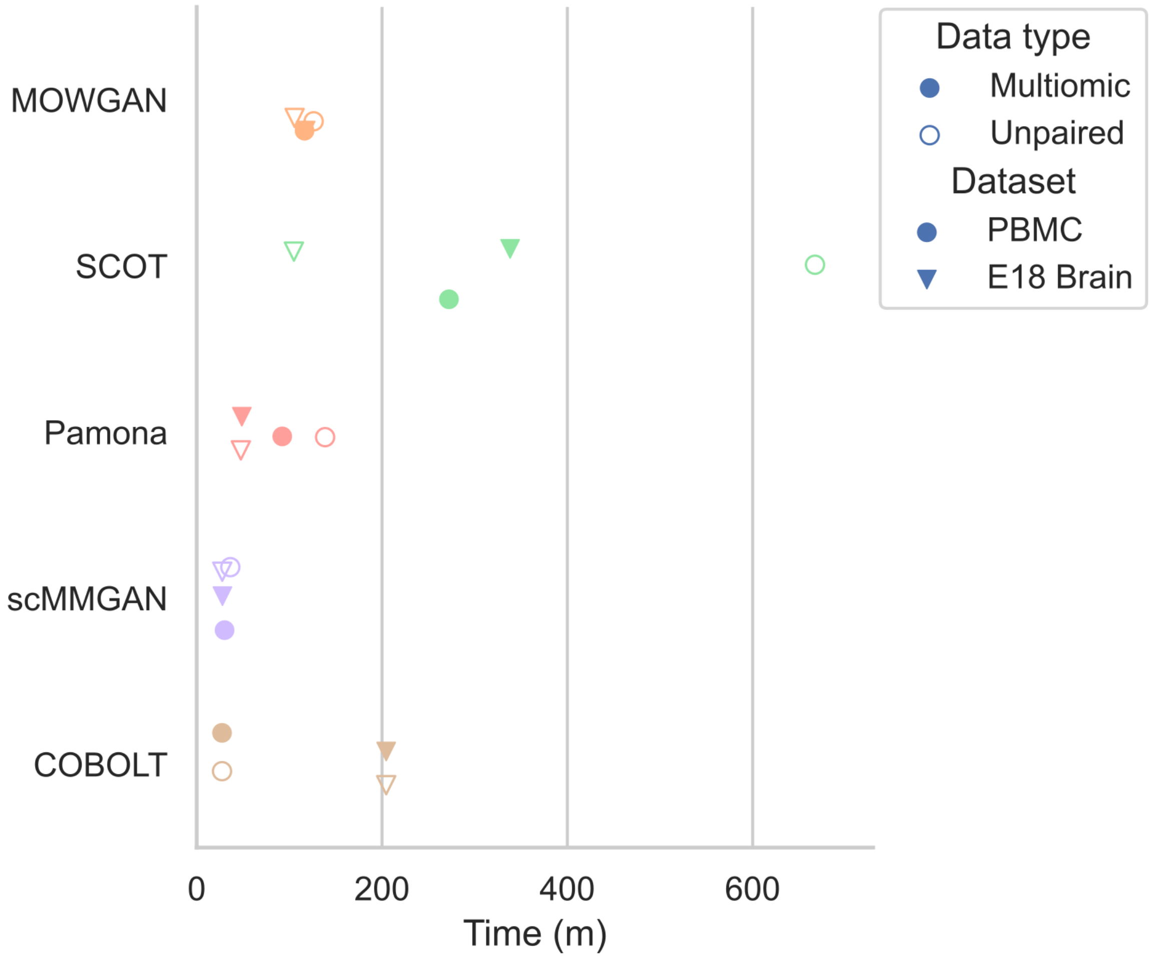

Fig. S3 Graph showing the running times (in minutes) registered for all tools used in benchmark on four datasets. Data were registered on a machine using 12 CPU and 30 Gb RAM. scMMGAN and MOWGAN were run with the support of 1 NVIDIA A100 GPU. Running times for paired and unpaired datasets for COBOLT are reported equal as COBOLT runs once using both datasets.

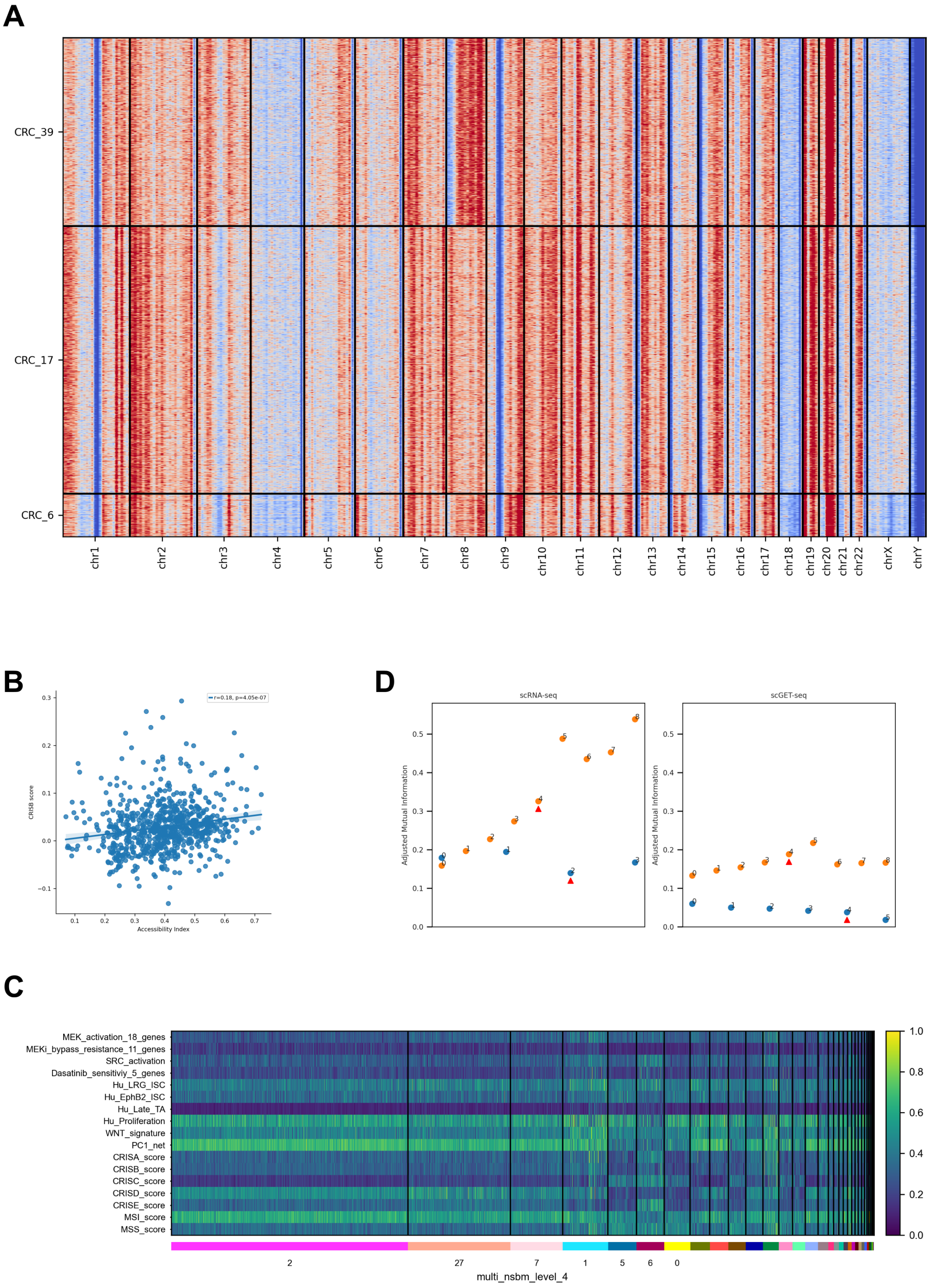

Fig. S4 (A) Segmentation profiles of PDO used in the manuscript at 1 Mb resolution. Each row represents a single cell, segmentation values are colored from red (amplification) to blue (deletion). (B) Scatterplot representing the correlation between Chromatin Accessibility Score, calculated on scGET-seq data, and CRIS-B signature score, calculated on scRNA-seq data. Each point represents the median value of a score in cell groups at level 0 of the NSBM hierarchy. (C) Profile of gene signatures described for drug resistance and phenotypes and CRC subtypes. The heatmap reports the score of each signature in cell groups found in integrated data. (D) Profile of Adjusted Mutual Information (AMI) between PDO identity and cell groups identified using the Nested Stochastic Block Models. Orange dots indicate the groups from the integrated data, blue dots indicate groups identified in single modalities. The number near each dot indicates the level of the NSBM hierarchy. The red triangle indicates the level used for the analysis described in the manuscript.

Supplementary Tables

Table S1 MOWGAN performance in prediction of cell types in different datasets. For all experiments we reported weighted and unweighted accuracy (see methods) and F1-score values. Accuracy values are also displayed in Figure 2B in the main text.

Dataset	Omic	Source	Data type	Weighted Accuracy	Accuracy	Weighted F1	F1	AMI
PBMC	RNA	Reference	Multiomic	0.7428	0.7959	0.6473	0.5833	0.6912
			Unpaired	0.7480	0.7755	0.6410	0.5600	NA
		MOWGAN	Multiomic	0.6885	0.7551	0.6027	0.5385	0.6301
			Unpaired	0.7681	0.7959	0.6633	0.5833	0.5856
	ATAC	Reference	Multiomic	0.7779	0.7347	0.5698	0.5185	0.6912
			Unpaired	0.6969	0.6939	0.5521	0.4828	NA
		MOWGAN	Multiomic	0.7679	0.7551	0.5795	0.5385	0.6184
			Unpaired	0.6284	0.6327	0.4982	0.4375	0.5088
E18 Brain	RNA	Reference	Multiomic	0.7849	0.8000	0.5195	0.4762	0.4479
			Unpaired	0.5313	0.6091	0.3005	0.3175	NA
		MOWGAN	Multiomic	0.6802	0.7273	0.4145	0.4000	0.4863
			Unpaired	0.5223	0.6091	0.3036	0.3175	0.3939
	ATAC	Reference	Multiomic	0.8297	0.8182	0.5874	0.5000	0.4479
			Unpaired	0.9703	0.9636	0.8729	0.8333	NA
		MOWGAN	Multiomic	0.7513	0.7879	0.4748	0.4615	0.4225
			Unpaired	0.8597	0.8364	0.5491	0.5263	0.4878

Table S2 Performance of multiple tools in integration of four datasets (PBMC and EMB). Homogeneity, completeness and V-measure were evaluated between cell types and cell clusters. We report one value for COBOLT for each metric in paired data as COBOLT produces a single embedding for paired data. LISI scores were calculated on the input embedding (PCA) and the embedding generated by the tool. In addition, the table include Adjusted Mutual Information (AMI) between cell clusters in datasets and cell clusters after each tool is applied. V-measure, LISI and AMI are reported in Figure 3 in the main text.

		**PBMC_1**		**PBMC_2**		**EMB_1**		**EMB_2**	
		**RNA**	**ATAC**	**RNA**	**ATAC**	**RNA**	**ATAC**	**RNA**	**ATAC**
Reference	Homogeneity	0.9399	0.8147	0.9424	0.7936	0.4208	0.2522	0.3247	0.4125
	Completeness	0.7353	0.5120	0.5269	0.3371	0.3638	0.2667	0.2519	0.3825
	V measure	0.8251	0.6288	0.6759	0.4732	0.3903	0.2593	0.2837	0.3969
	LISI*	1.2050	1.3538	1.2050	1.3538	1.2605	1.3889	1.2605	1.3889
	AMI	0.6912		NA		0.4479		NA	
MOWGAN	Homogeneity	0.8095	0.8537	0.7862	0.8141	0.5988	0.5572	0.5486	0.2151
	Completeness	0.3436	0.3589	0.2535	0.3113	0.4630	0.4322	0.3242	0.4923
	V measure	0.4824	0.5054	0.3833	0.4504	0.5222	0.4868	0.4075	0.5602
	LISI	1.6364	1.6410	1.3546	1.4376	1.7034	1.7558	1.6049	1.8287
	AMI	0.3734		0.3949		0.2444		0.2151	
SCOT	Homogeneity	0.9128	0.7467	0.9452	0.6885	0.4224	0.3118	0.3651	0.4224
	Completeness	0.4063	0.2531	0.4568	0.2212	0.3119	0.2144	0.2299	0.2697
	V measure	0.5623	0.3781	0.6159	0.3348	0.3588	0.2541	0.2821	0.3292
	LISI	1.0084	1.0117	1.0758	1.0578	1.0059	1.0081	1.0175	1.0148
	AMI	0.3962		-0.0009		0.3378		0.0055	
Pamona	Homogeneity	0.9177	0.8568	0.9447	0.7123	0.4232	0.3651	0.4026	0.4913
	Completeness	0.2779	0.2585	0.2964	0.2165	0.2627	0.2288	0.2023	0.2944
	V measure	0.4266	0.3971	0.4512	0.3321	0.3242	0.2813	0.2693	0.3682
	LISI	1.0312	1.0133	1.0267	1.0244	1.0686	1.0442	1.0550	1.0624
	AMI	0.2783		0.0017		0.2781		-0.0001	
scMMGAN	Homogeneity	0.8058	0.7486	0.9190	0.7416	0.3757	0.3105	0.3360	0.4449
	Completeness	0.3524	0.3194	0.3711	0.3091	0.2815	0.2320	0.1926	0.3116
	V measure	0.4904	0.4477	0.5287	0.4364	0.3219	0.2656	0.2449	0.3665
	LISI	1.3392	1.4612	1.3698	1.1913	1.4996	1.7075	1.5247	1.5743
	AMI	0.4092		-0.1925		0.3377		0.0144	
COBOLT	Homogeneity	0.8754		0.9471	0.0154	0.4019		0.3098	0.4038
	Completeness	0.4167		0.4325	0.0066	0.3068		0.1972	0.2863
	V measure	0.5646		0.5938	0.0093	0.3480		0.2410	0.3351
	LISI	1.1349	1.1219	1.0672	1.0769	1.1418	1.1925	1.1635	1.1337
	AMI	1.0000		0.0103		1.0000		0.0037	

Table S3 Running times (in seconds) registered for all tools used in benchmark on four datasets. Data were registered on a machine using 12 CPU and 30 Gb RAM. scMMGAN and MOWGAN were run with the support of 1 NVIDIA A100 GPU. Running times for paired and unpaired datasets for COBOLT are reported equal as COBOLT runs once using both datasets.

Dataset	Method	Data type	Time
PBMC	MOWGAN	Multiomic	6983.68338
		Unpaired	7572.48252
	SCOT	Multiomic	16334.0461
		Unpaired	40036.0021
	Pamona	Multiomic	5537.30978
		Unpaired	8313.14911
	scMMGAN	Multiomic	1804.53208
		Unpaired	2175.7037
	COBOLT	Multiomic	1639.14082
		Unpaired	1639.14082
E18 Brain	MOWGAN	Multiomic	7056.55783
		Unpaired	6339.46865
	SCOT	Multiomic	20293.1074
		Unpaired	6293.90432
	Pamona	Multiomic	2920.17263
		Unpaired	2858.32188
	scMMGAN	Multiomic	1666.08671
		Unpaired	1654.20241
	COBOLT	Multiomic	12269.5337
		Unpaired	12269.5337

Table S4 Performance of integration of high number of modalities using MOWGAN. The table reports multiple scores calculated after four layer integration (RNA, ATAC, ADP and H3K27me3) of PBMC data, evaluated on the ability to transfer cell type annotation. The first three columns refer to label transfer from RNA to the remaining modalities. The other three columns report the values obtained by transferring labels from any other modality to RNA.

	**Bridge from RNA**			**Bridge to RNA**		
	**ATAC**	**ADT**	**H3K27me3**	**ATAC**	**ADT**	**H3K27me3**
Accuracy	0.8287	0.8050	0.6568	0.9175	0.8805	0.8492
AMI	0.6457	0.6150	0.3734	0.7707	0.7562	0.6805
Homogeneity	0.6981	0.6789	0.4122	0.7835	0.7628	0.6650
Completeness	0.6011	0.5626	0.3420	0.7586	0.7501	0.6971
V-measure	0.6460	0.6153	0.3739	0.7708	0.7564	0.6807

Table S5 Characterization of the PDO dataset. The table reports clinical features of three PDOs (CRC6, CRC17 and CRC39) and the mutational status of cancer genes commonly profiled in CRC. We also report the number of single cells analyzed in the corresponding scRNA-seq and scGET-seq datasets.

	**Clinical features**		**Mutations**				**Number of single cells**	
**PDO ID**	**Metastatis**	**Chemoterapy**	**APC**	**KRAS**	**TP53**	**PIK3CA**	**RNA**	**GET**
6 CRC	Yes	Yes	Yes	No	Yes	No	517	4310
17 CRC	Yes	Yes	Yes	Yes	Yes	No	4864	4998
39 CRC	No	No	No	Yes	Yes	Yes	1105	5000

Table S6 Correlation between TF activity, computed on scRNA-seq data, and TBA scores, computed on scGET-seq data, in PDO datasets. We report Pearson's r value and the associated p-value for transcription factors that were present in both analyses. Data are sorted by decreasing correlation.

Transcription Factor	HOCOMOCO Motif	Pearson r	p-value
SPI1	SPI1_HUMAN.H11MO.0.A	0.421245087	3.11E-33
REST	REST_HUMAN.H11MO.0.A	0.393502537	7.46E-29
NFE2L2	NF2L2_HUMAN.H11MO.0.A	0.391044209	1.74E-28
EHF	EHF_HUMAN.H11MO.0.B	0.385574686	1.12E-27
NR2F1	COT1_HUMAN.H11MO.0.C	0.384181357	1.79E-27
ESR2	ESR2_HUMAN.H11MO.0.A	0.382145243	3.54E-27
NR2F2	COT2_HUMAN.H11MO.1.A	0.381974802	3.75E-27
NR2F1	COT1_HUMAN.H11MO.1.C	0.381893199	3.85E-27
NR2F2	COT2_HUMAN.H11MO.0.A	0.381536847	4.33E-27
HNF1A	HNF1A_HUMAN.H11MO.0.C	0.372505058	8.34E-26
HNF4G	HNF4G_HUMAN.H11MO.0.B	0.370187788	1.75E-25
FOS	FOS_HUMAN.H11MO.0.A	0.368903784	2.64E-25
KLF6	KLF6_HUMAN.H11MO.0.A	0.36888196	2.66E-25
TBP	TBP_HUMAN.H11MO.0.A	0.363464664	1.47E-24
STAT2	STAT2_HUMAN.H11MO.0.A	0.350943136	6.71E-23
RELB	RELB_HUMAN.H11MO.0.C	0.335520672	5.92E-21
MAFK	MAFK_HUMAN.H11MO.1.A	0.328321856	4.40E-20
MAFK	MAFK_HUMAN.H11MO.0.A	0.327956449	4.86E-20
NFKB1	NFKB1_HUMAN.H11MO.1.B	0.32113037	3.09E-19
STAT3	STAT3_HUMAN.H11MO.0.A	0.31801828	7.08E-19
NR1H2	NR1H2_HUMAN.H11MO.0.D	0.313185832	2.51E-18
SOX9	SOX9_HUMAN.H11MO.0.B	0.291172514	6.03E-16
HNF1B	HNF1B_HUMAN.H11MO.0.A	0.290291183	7.44E-16
HNF1B	HNF1B_HUMAN.H11MO.1.A	0.289720345	8.52E-16
ESRRA	ERR1_HUMAN.H11MO.0.A	0.289691445	8.58E-16
CDX2	CDX2_HUMAN.H11MO.0.A	0.280007326	8.15E-15
PRDM1	PRDM1_HUMAN.H11MO.0.A	0.271878968	5.05E-14
WT1	WT1_HUMAN.H11MO.0.C	0.263730406	2.95E-13
SP2	SP2_HUMAN.H11MO.0.A	0.263196152	3.31E-13
PRDM14	PRD14_HUMAN.H11MO.0.A	0.260999231	5.27E-13
FOSL1	FOSL1_HUMAN.H11MO.0.A	0.25988972	6.65E-13
TWIST1	TWST1_HUMAN.H11MO.0.A	0.256339095	1.39E-12
RFX5	RFX5_HUMAN.H11MO.0.A	0.25361595	2.44E-12
MYB	MYB_HUMAN.H11MO.0.A	0.253069702	2.72E-12
TWIST1	TWST1_HUMAN.H11MO.1.A	0.247134455	8.97E-12
RFX5	RFX5_HUMAN.H11MO.1.A	0.246391721	1.04E-11
KLF3	KLF3_HUMAN.H11MO.0.B	0.244820832	1.42E-11
WT1	WT1_HUMAN.H11MO.1.B	0.23884047	4.51E-11
SP2	SP2_HUMAN.H11MO.1.B	0.238174786	5.13E-11
STAT1	STAT1_HUMAN.H11MO.0.A	0.236407301	7.17E-11
STAT1	STAT1_HUMAN.H11MO.1.A	0.236129892	7.56E-11
RUNX3	RUNX3_HUMAN.H11MO.0.A	0.235979593	7.77E-11
DUX4	DUX4_HUMAN.H11MO.0.A	0.23535725	8.74E-11
ELF3	ELF3_HUMAN.H11MO.0.A	0.234533537	1.02E-10
MAZ	MAZ_HUMAN.H11MO.0.A	0.233434685	1.25E-10
MITF	MITF_HUMAN.H11MO.0.A	0.232061981	1.62E-10

FOSL2	FOSL2_HUMAN.H11MO.0.A	0.23134952	1.85E-10
NFKB2	NFKB2_HUMAN.H11MO.0.B	0.229384959	2.65E-10
ETV4	ETV4_HUMAN.H11MO.0.B	0.222413671	9.30E-10
SNAI1	SNAI1_HUMAN.H11MO.0.C	0.221301081	1.13E-09
TEAD2	TEAD2_HUMAN.H11MO.0.D	0.21956325	1.54E-09
GATA1	GATA1_HUMAN.H11MO.1.A	0.217988594	2.02E-09
YY1	TYY1_HUMAN.H11MO.0.A	0.216067046	2.82E-09
GATA1	GATA1_HUMAN.H11MO.0.A	0.211606372	6.01E-09
IRF8	IRF8_HUMAN.H11MO.0.B	0.202076782	2.88E-08
CEBPG	CEBPG_HUMAN.H11MO.0.B	0.195342502	8.31E-08
TP63	P63_HUMAN.H11MO.0.A	0.186782096	3.04E-07
ATF3	ATF3_HUMAN.H11MO.0.A	0.182966233	5.32E-07
E2F6	E2F6_HUMAN.H11MO.0.A	0.182403078	5.77E-07
TP63	P63_HUMAN.H11MO.1.A	0.181718225	6.37E-07
CREB1	CREB1_HUMAN.H11MO.0.A	0.179856615	8.31E-07
GATA6	GATA6_HUMAN.H11MO.0.A	0.17590691	1.45E-06
RUNX1	RUNX1_HUMAN.H11MO.0.A	0.174729078	1.71E-06
EOMES	EOMES_HUMAN.H11MO.0.D	0.171896982	2.52E-06
ONECUT1	HNF6_HUMAN.H11MO.0.B	0.17066398	2.98E-06
ZBTB33	KAISO_HUMAN.H11MO.1.A	0.170042628	3.24E-06
ZBTB33	KAISO_HUMAN.H11MO.2.A	0.169742743	3.37E-06
ZBTB33	KAISO_HUMAN.H11MO.0.A	0.16904195	3.71E-06
BACH2	BACH2_HUMAN.H11MO.0.A	0.168230067	4.13E-06
TCF7	TCF7_HUMAN.H11MO.0.A	0.166159644	5.44E-06
IRF2	IRF2_HUMAN.H11MO.0.A	0.160131656	1.19E-05
AR	ANDR_HUMAN.H11MO.2.A	0.152674282	3.00E-05
TCF3	TFE2_HUMAN.H11MO.0.A	0.147535546	5.54E-05
PPARA	PPARA_HUMAN.H11MO.0.B	0.141353589	0.000113046
AR	ANDR_HUMAN.H11MO.1.A	0.139007976	0.000147028
AR	ANDR_HUMAN.H11MO.0.A	0.138499034	0.000155572
PPARA	PPARA_HUMAN.H11MO.1.B	0.138439225	0.000156606
POU2F1	PO2F1_HUMAN.H11MO.0.C	0.137988527	0.000164609
GRHL2	GRHL2_HUMAN.H11MO.0.A	0.137907599	0.000166086
ELF5	ELF5_HUMAN.H11MO.0.A	0.128201503	0.000468116
SNAI2	SNAI2_HUMAN.H11MO.0.A	0.124315642	0.000695146
MAFG	MAFG_HUMAN.H11MO.0.A	0.122027443	0.000872879
ESR1	ESR1_HUMAN.H11MO.1.A	0.121151224	0.000951435
PAX6	PAX6_HUMAN.H11MO.0.C	0.119494784	0.001118066
ZEB1	ZEB1_HUMAN.H11MO.0.A	0.118007308	0.00129024
NFIC	NFIC_HUMAN.H11MO.0.A	0.116169894	0.001536555
MEF2B	MEF2B_HUMAN.H11MO.0.A	0.11594825	0.001569024
NRF1	NRF1_HUMAN.H11MO.0.A	0.102444349	0.005249533
MXI1	MXI1_HUMAN.H11MO.0.A	0.100827027	0.006014058
MXI1	MXI1_HUMAN.H11MO.1.A	0.100142098	0.00636697
GABPA	GABPA_HUMAN.H11MO.0.A	0.098922274	0.007041805
RARA	RARA_HUMAN.H11MO.1.A	0.092769995	0.011520268
FOXK1	FOXK1_HUMAN.H11MO.0.A	0.089714925	0.014567222
ETS2	ETS2_HUMAN.H11MO.0.B	0.089296891	0.015035028
HBP1	HBP1_HUMAN.H11MO.0.D	0.085898337	0.019353972
KLF9	KLF9_HUMAN.H11MO.0.C	0.085815602	0.019471389
KLF1	KLF1_HUMAN.H11MO.0.A	0.085578557	0.019811249

RXRG	RXRG_HUMAN.H11MO.0.B	0.085454571	0.019991061
KLF4	KLF4_HUMAN.H11MO.0.A	0.083203819	0.023511317
MAZ	MAZ_HUMAN.H11MO.1.A	0.073815849	0.04456756
HINFP	HINFP_HUMAN.H11MO.0.C	0.073442474	0.045659761
TCF4	ITF2_HUMAN.H11MO.0.C	0.073074398	0.046758403
IKZF1	IKZF1_HUMAN.H11MO.0.C	0.072300502	0.049140869
TFAP2A	AP2A_HUMAN.H11MO.0.A	0.067675282	0.065590592
CUX1	CUX1_HUMAN.H11MO.0.C	0.062720077	0.087985618
E2F3	E2F3_HUMAN.H11MO.0.A	0.061401907	0.094880779
MAF	MAF_HUMAN.H11MO.0.A	0.059116702	0.107851812
FOXA1	FOXA1_HUMAN.H11MO.0.A	0.056004571	0.127723706
TAL1	TAL1_HUMAN.H11MO.0.A	0.055649995	0.130157368
CTCFL	CTCFL_HUMAN.H11MO.0.A	0.055457523	0.131493376
ELK1	ELK1_HUMAN.H11MO.0.B	0.055310723	0.132519475
VDR	VDR_HUMAN.H11MO.1.A	0.054409665	0.138953749
TAL1	TAL1_HUMAN.H11MO.1.A	0.053244762	0.147624665
MAX	MAX_HUMAN.H11MO.0.A	0.052411635	0.154074915
BCL11A	BC11A_HUMAN.H11MO.0.A	0.052340192	0.154637841
HSF1	HSF1_HUMAN.H11MO.1.A	0.051900355	0.158137876
HOXA9	HXA9_HUMAN.H11MO.0.B	0.048526359	0.187002451
TCF7L2	TF7L2_HUMAN.H11MO.0.A	0.047901268	0.192751692
GATA3	GATA3_HUMAN.H11MO.0.A	0.047437078	0.197104193
SPIB	SPIB_HUMAN.H11MO.0.A	0.044155875	0.229930714
SOX13	SOX13_HUMAN.H11MO.0.D	0.043955588	0.232053214
RARG	RARG_HUMAN.H11MO.1.B	0.040618821	0.269468733
PDX1	PDX1_HUMAN.H11MO.1.A	0.039093586	0.28787785
FOXA2	FOXA2_HUMAN.H11MO.0.A	0.038628226	0.293659322
NR1H4	NR1H4_HUMAN.H11MO.0.B	0.038339759	0.297281903
ZNF263	ZN263_HUMAN.H11MO.0.A	0.037698312	0.305443655
MAFG	MAFG_HUMAN.H11MO.1.A	0.037066612	0.31362502
PROX1	PROX1_HUMAN.H11MO.0.D	0.036729739	0.318046269
NR4A1	NR4A1_HUMAN.H11MO.0.A	0.034984463	0.341601084
JUN	JUN_HUMAN.H11MO.0.A	0.03460246	0.346901691
FOXP1	FOXP1_HUMAN.H11MO.0.A	0.034247224	0.351877498
HSF1	HSF1_HUMAN.H11MO.0.A	0.034200596	0.35253396
RELA	TF65_HUMAN.H11MO.0.A	0.033465541	0.362984472
HES1	HES1_HUMAN.H11MO.0.D	0.032412021	0.378296273
ETV1	ETV1_HUMAN.H11MO.0.A	0.030570072	0.40600362
FLI1	FLI1_HUMAN.H11MO.0.A	0.030510704	0.406916323
JUNB	JUNB_HUMAN.H11MO.0.A	0.0293828	0.424487237
FLI1	FLI1_HUMAN.H11MO.1.A	0.027349962	0.457249156
PDX1	PDX1_HUMAN.H11MO.0.A	0.027136525	0.460769316
GATA2	GATA2_HUMAN.H11MO.1.A	0.026321342	0.474352103
E2F5	E2F5_HUMAN.H11MO.0.B	0.025252756	0.492484744
ATF2	ATF2_HUMAN.H11MO.0.B	0.024189268	0.510892737
ATF2	ATF2_HUMAN.H11MO.2.C	0.023928988	0.51545198
GFI1B	GFI1B_HUMAN.H11MO.0.A	0.023739579	0.518782991
ATF2	ATF2_HUMAN.H11MO.1.B	0.023699126	0.519495849
GATA2	GATA2_HUMAN.H11MO.0.A	0.021954799	0.550706709
ATF7	ATF7_HUMAN.H11MO.0.D	0.020716103	0.573415847
USF2	USF2_HUMAN.H11MO.0.A	0.018249807	0.61990403

SOX10	SOX10_HUMAN.H11MO.1.A	0.017400436	0.636285296
HMBOX1	HMBX1_HUMAN.H11MO.0.D	0.014871587	0.686092806
SOX10	SOX10_HUMAN.H11MO.0.B	0.012571161	0.732622345
TEAD1	TEAD1_HUMAN.H11MO.0.A	0.010639468	0.77247459
CEBPD	CEBPD_HUMAN.H11MO.0.C	0.008656328	0.814019948
NR2C2	NR2C2_HUMAN.H11MO.0.B	0.007407374	0.84046336
MYOD1	MYOD1_HUMAN.H11MO.1.A	0.006248329	0.865165667
KLF5	KLF5_HUMAN.H11MO.0.A	0.005895969	0.872702687
FOXO4	FOXO4_HUMAN.H11MO.0.C	0.005523496	0.88068245
ZKSCAN1	ZKSC1_HUMAN.H11MO.0.B	0.002322322	0.949678904
TCF12	HTF4_HUMAN.H11MO.0.A	0.001657825	0.964065836
MYOD1	MYOD1_HUMAN.H11MO.0.A	0.001587001	0.965600011
RUNX2	RUNX2_HUMAN.H11MO.0.A	0.001346409	0.970812579
MYC	MYC_HUMAN.H11MO.0.A	0.000224695	0.995128004
BACH1	BACH1_HUMAN.H11MO.0.A	-0.00056917	0.987659285
CLOCK	CLOCK_HUMAN.H11MO.0.C	-0.002139458	0.953636635
ZBTB7A	ZBT7A_HUMAN.H11MO.0.A	-0.005591839	0.879217367
SOX11	SOX11_HUMAN.H11MO.0.D	-0.006443855	0.860988566
ZFX	ZFX_HUMAN.H11MO.0.A	-0.010298526	0.779574496
PGR	PRGR_HUMAN.H11MO.1.A	-0.01216392	0.74096846
IRF9	IRF9_HUMAN.H11MO.0.C	-0.014721368	0.689097481
NANOG	NANOG_HUMAN.H11MO.1.B	-0.018139216	0.622026525
NANOG	NANOG_HUMAN.H11MO.0.A	-0.020517754	0.577092935
TP53	P53_HUMAN.H11MO.1.A	-0.020718662	0.57336847
BHLHE40	BHE40_HUMAN.H11MO.0.A	-0.021261936	0.563354545
TP53	P53_HUMAN.H11MO.0.A	-0.02154589	0.558154233
TFAP4	TFAP4_HUMAN.H11MO.0.A	-0.022460216	0.541569411
MYBL2	MYBB_HUMAN.H11MO.0.D	-0.022558244	0.53980598
ZNF263	ZN263_HUMAN.H11MO.1.A	-0.023573587	0.521711285
BATF	BATF_HUMAN.H11MO.0.A	-0.027428231	0.455962075
ELF1	ELF1_HUMAN.H11MO.0.A	-0.027950751	0.447421888
STAT5B	STA5B_HUMAN.H11MO.0.A	-0.028890711	0.432289775
HNF4A	HNF4A_HUMAN.H11MO.0.A	-0.030932898	0.400452236
RFX2	RFX2_HUMAN.H11MO.1.A	-0.034470409	0.348746091
RFX2	RFX2_HUMAN.H11MO.0.A	-0.034987423	0.341560214
MAFB	MAFB_HUMAN.H11MO.0.B	-0.038306312	0.297703852
TGIF2	TGIF2_HUMAN.H11MO.0.D	-0.038901867	0.290250357
ATF6	ATF6A_HUMAN.H11MO.0.B	-0.039112272	0.287647316
BATF	BATF_HUMAN.H11MO.1.A	-0.042141267	0.251913899
OTX2	OTX2_HUMAN.H11MO.0.A	-0.044119654	0.230313531
NR1H4	NR1H4_HUMAN.H11MO.1.B	-0.045897291	0.212055563
IRF1	IRF1_HUMAN.H11MO.0.A	-0.049041698	0.182358515
RARA	RARA_HUMAN.H11MO.2.A	-0.050692316	0.168058762
RARA	RARA_HUMAN.H11MO.0.A	-0.050747867	0.167592558
ATF1	ATF1_HUMAN.H11MO.0.B	-0.053444407	0.146110006
SIX2	SIX2_HUMAN.H11MO.0.A	-0.057014667	0.120984241
VDR	VDR_HUMAN.H11MO.0.A	-0.061868942	0.092389959
PPARG	PPARG_HUMAN.H11MO.0.A	-0.062027733	0.091555127
RXRB	RXRB_HUMAN.H11MO.0.C	-0.062889131	0.087131262
MAF	MAF_HUMAN.H11MO.1.B	-0.064477451	0.079426503
GATA4	GATA4_HUMAN.H11MO.0.A	-0.065230433	0.075972102

ATF4	ATF4_HUMAN.H11MO.0.A	-0.066800355	0.069162935
RARG	RARG_HUMAN.H11MO.2.D	-0.067332833	0.066970311
RARG	RARG_HUMAN.H11MO.0.B	-0.067756012	0.065268764
TP73	P73_HUMAN.H11MO.1.A	-0.068888815	0.060888348
PGR	PRGR_HUMAN.H11MO.0.A	-0.069251915	0.059536819
SMAD1	SMAD1_HUMAN.H11MO.0.D	-0.069941273	0.057039224
TP73	P73_HUMAN.H11MO.0.A	-0.070990548	0.05340504
EBF1	COE1_HUMAN.H11MO.0.A	-0.071217542	0.052644777
SREBF2	SRBP2_HUMAN.H11MO.0.B	-0.072343106	0.049007116
CREB3L1	CR3L1_HUMAN.H11MO.0.D	-0.07239341	0.048849583
TEAD4	TEAD4_HUMAN.H11MO.0.A	-0.075804063	0.039115327
THAP11	THA11_HUMAN.H11MO.0.B	-0.076653969	0.036963292
ERG	ERG_HUMAN.H11MO.0.A	-0.077312902	0.035364836
LEF1	LEF1_HUMAN.H11MO.0.A	-0.077586367	0.034718955
TFDP1	TFDP1_HUMAN.H11MO.0.C	-0.083556514	0.022926506
NR5A2	NR5A2_HUMAN.H11MO.0.B	-0.08525431	0.020284497
EPAS1	EPAS1_HUMAN.H11MO.0.B	-0.087423152	0.017297874
STAT4	STAT4_HUMAN.H11MO.0.A	-0.087828499	0.016784544
AHR	AHR_HUMAN.H11MO.0.B	-0.089947267	0.014312794
ZFX	ZFX_HUMAN.H11MO.1.A	-0.091056369	0.013151418
NFATC1	NFAC1_HUMAN.H11MO.1.B	-0.092163261	0.012076075
MEIS1	MEIS1_HUMAN.H11MO.1.B	-0.092886136	0.011416497
ARID3A	ARI3A_HUMAN.H11MO.0.D	-0.093464774	0.010911734
TBX21	TBX21_HUMAN.H11MO.0.A	-0.095830062	0.009048245
SP3	SP3_HUMAN.H11MO.0.B	-0.095981479	0.008939235
HOXB13	HXB13_HUMAN.H11MO.0.A	-0.097877699	0.007669896
ZEB2	ZEB1_HUMAN.H11MO.0.A	-0.09858596	0.007238804
FOXM1	FOXM1_HUMAN.H11MO.0.A	-0.099015427	0.006988097
E2F1	E2F1_HUMAN.H11MO.0.A	-0.100355146	0.006255253
CREB3	CREB3_HUMAN.H11MO.0.D	-0.102570235	0.005193871
BHLHE22	BHE22_HUMAN.H11MO.0.D	-0.1054423	0.004060203
LYL1	LYL1_HUMAN.H11MO.0.A	-0.107328517	0.003442964
PBX2	PBX2_HUMAN.H11MO.0.C	-0.110646839	0.002560207
E2F2	E2F2_HUMAN.H11MO.0.B	-0.113246546	0.002018797
THAP1	THAP1_HUMAN.H11MO.0.C	-0.114160273	0.001854927
NFIC	NFIC_HUMAN.H11MO.1.A	-0.116598376	0.00147553
NFATC1	NFAC1_HUMAN.H11MO.0.B	-0.117966964	0.001295233
GLI2	GLI2_HUMAN.H11MO.0.D	-0.118609653	0.00121777
SOX2	SOX2_HUMAN.H11MO.1.A	-0.118894194	0.001184858
SMAD3	SMAD3_HUMAN.H11MO.0.B	-0.119884649	0.001076587
SOX2	SOX2_HUMAN.H11MO.0.A	-0.120924001	0.000972847
MEIS1	MEIS1_HUMAN.H11MO.0.A	-0.122268193	0.000852371
ZNF384	ZN384_HUMAN.H11MO.0.C	-0.127228486	0.000517372
ELK4	ELK4_HUMAN.H11MO.0.A	-0.128996243	0.000431162
PPARG	PPARG_HUMAN.H11MO.1.A	-0.132225998	0.000307205
PKNOX1	PKNX1_HUMAN.H11MO.0.B	-0.132654318	0.000293532
REL	REL_HUMAN.H11MO.0.B	-0.13323698	0.000275843
CREM	CREM_HUMAN.H11MO.0.C	-0.136935319	0.000184833
ZNF143	ZN143_HUMAN.H11MO.0.A	-0.137228899	0.000178973
RXRA	RXRA_HUMAN.H11MO.1.A	-0.143200206	9.17E-05
POU4F2	PO4F2_HUMAN.H11MO.0.D	-0.147248939	5.73E-05

CEBPB	CEBPB_HUMAN.H11MO.0.A	-0.147334808	5.68E-05
FOXO3	FOXO3_HUMAN.H11MO.0.B	-0.147411112	5.63E-05
MEIS2	MEIS2_HUMAN.H11MO.0.B	-0.149019901	4.65E-05
HIF1A	HIF1A_HUMAN.H11MO.0.C	-0.152357898	3.12E-05
XBP1	XBP1_HUMAN.H11MO.0.D	-0.154234405	2.48E-05
IRF4	IRF4_HUMAN.H11MO.0.A	-0.156196385	1.95E-05
POU2F2	PO2F2_HUMAN.H11MO.0.A	-0.158351441	1.49E-05
ETV6	ETV6_HUMAN.H11MO.0.D	-0.159733944	1.25E-05
BCL6	BCL6_HUMAN.H11MO.0.A	-0.160121454	1.19E-05
FOXJ2	FOXJ2_HUMAN.H11MO.0.C	-0.163570939	7.63E-06
SP1	SP1_HUMAN.H11MO.0.A	-0.166629399	5.11E-06
RBPJ	SUH_HUMAN.H11MO.0.A	-0.16747417	4.57E-06
RXRA	RXRA_HUMAN.H11MO.0.A	-0.16831612	4.09E-06
ZNF274	ZN274_HUMAN.H11MO.0.A	-0.16893019	3.76E-06
USF1	USF1_HUMAN.H11MO.0.A	-0.16987834	3.31E-06
SP1	SP1_HUMAN.H11MO.1.A	-0.173007858	2.17E-06
ARNT	ARNT_HUMAN.H11MO.0.B	-0.174733496	1.71E-06
ZNF740	ZN740_HUMAN.H11MO.0.D	-0.176927231	1.26E-06
POU5F1	PO5F1_HUMAN.H11MO.1.A	-0.180459137	7.63E-07
NFYB	NFYB_HUMAN.H11MO.0.A	-0.1810335	7.02E-07
POU5F1	PO5F1_HUMAN.H11MO.0.A	-0.185288811	3.79E-07
SMAD4	SMAD4_HUMAN.H11MO.0.B	-0.190842583	1.66E-07
ESR1	ESR1_HUMAN.H11MO.0.A	-0.191915009	1.41E-07
ZBED1	ZBED1_HUMAN.H11MO.0.D	-0.196109742	7.38E-08
STAT6	STAT6_HUMAN.H11MO.0.B	-0.196476802	6.97E-08
TFAP2C	AP2C_HUMAN.H11MO.0.A	-0.198956549	4.72E-08
MAFF	MAFF_HUMAN.H11MO.1.B	-0.199283633	4.49E-08
MAFF	MAFF_HUMAN.H11MO.0.B	-0.199785199	4.14E-08
STAT5A	STA5A_HUMAN.H11MO.0.A	-0.200666629	3.60E-08
MBD2	MBD2_HUMAN.H11MO.0.B	-0.204056803	2.09E-08
RFX1	RFX1_HUMAN.H11MO.1.B	-0.20789212	1.12E-08
RFX1	RFX1_HUMAN.H11MO.0.B	-0.208604613	9.92E-09
ASCL1	ASCL1_HUMAN.H11MO.0.A	-0.215104345	3.32E-09
FOXO1	FOXO1_HUMAN.H11MO.0.A	-0.218102824	1.98E-09
NR3C1	GCR_HUMAN.H11MO.0.A	-0.218506862	1.85E-09
NEUROD1	NDF1_HUMAN.H11MO.0.A	-0.219059164	1.68E-09
E2F4	E2F4_HUMAN.H11MO.0.A	-0.219367662	1.59E-09
MEF2A	MEF2A_HUMAN.H11MO.0.A	-0.220210198	1.37E-09
NR1H3	NR1H3_HUMAN.H11MO.1.B	-0.220412393	1.32E-09
E2F4	E2F4_HUMAN.H11MO.1.A	-0.22060948	1.28E-09
JUND	JUND_HUMAN.H11MO.0.A	-0.221772289	1.04E-09
PAX5	PAX5_HUMAN.H11MO.0.A	-0.224663956	6.23E-10
NR3C1	GCR_HUMAN.H11MO.1.A	-0.224841712	6.03E-10
NFE2L1	NF2L1_HUMAN.H11MO.0.C	-0.233024504	1.35E-10
E2F7	E2F7_HUMAN.H11MO.0.B	-0.239040435	4.35E-11
SREBF1	SRBP1_HUMAN.H11MO.0.A	-0.239175572	4.23E-11
MEF2C	MEF2C_HUMAN.H11MO.0.A	-0.241148487	2.90E-11
KLF13	KLF13_HUMAN.H11MO.0.D	-0.245714081	1.19E-11
SRF	SRF_HUMAN.H11MO.0.A	-0.249611563	5.48E-12
NR1H3	NR1H3_HUMAN.H11MO.0.B	-0.257410884	1.12E-12
LHX2	LHX2_HUMAN.H11MO.0.A	-0.258349451	9.18E-13

NFE2	NFE2_HUMAN.H11MO.0.A	-0.258647215	8.62E-13
ETS1	ETS1_HUMAN.H11MO.0.A	-0.25869002	8.55E-13
HIC1	HIC1_HUMAN.H11MO.0.C	-0.263933427	2.83E-13
PBX3	PBX3_HUMAN.H11MO.1.A	-0.277065399	1.59E-14
NR2F6	NR2F6_HUMAN.H11MO.0.D	-0.277943007	1.30E-14
SOX9	SOX9_HUMAN.H11MO.1.B	-0.278255173	1.21E-14
PBX3	PBX3_HUMAN.H11MO.0.A	-0.288637054	1.10E-15
EGR1	EGR1_HUMAN.H11MO.0.A	-0.29298634	3.91E-16
ESR2	ESR2_HUMAN.H11MO.1.A	-0.29655916	1.65E-16
CEBPA	CEBPA_HUMAN.H11MO.0.A	-0.307448851	1.10E-17
MYCN	MYCN_HUMAN.H11MO.0.A	-0.30754667	1.07E-17
IRF3	IRF3_HUMAN.H11MO.0.B	-0.317035492	9.18E-19
NFYA	NFYA_HUMAN.H11MO.0.A	-0.317499787	8.12E-19
FOXP2	FOXP2_HUMAN.H11MO.0.C	-0.330247818	2.58E-20
CTCF	CTCF_HUMAN.H11MO.0.A	-0.342614744	7.78E-22
SP4	SP4_HUMAN.H11MO.1.A	-0.376730429	2.12E-26
SP4	SP4_HUMAN.H11MO.0.A	-0.385384766	1.20E-27
